# Ebola virus VP35 interacts non-covalently with ubiquitin chains to promote viral replication

**DOI:** 10.1371/journal.pbio.3002544

**Published:** 2024-02-29

**Authors:** Carlos A. Rodríguez-Salazar, Sarah van Tol, Olivier Mailhot, Maria Gonzalez-Orozco, Gabriel T. Galdino, Abbey N. Warren, Natalia Teruel, Padmanava Behera, Kazi Sabrina Afreen, Lihong Zhang, Terry L. Juelich, Jennifer K. Smith, María Inés Zylber, Alexander N. Freiberg, Rafael J. Najmanovich, Maria I. Giraldo, Ricardo Rajsbaum

**Affiliations:** 1 Department of Microbiology and Immunology, University of Texas Medical Branch, Galveston, Texas, United States of America; 2 Molecular Biology and Virology Laboratory, Faculty of Medicine and Health Sciences, Corporación Universitaria Empresarial Alexander von Humboldt, Armenia, Colombia; 3 Department of Pharmacology and Physiology, Faculty of Medicine, Université de Montréal, Montreal, Canada; 4 Center for Virus-Host-Innate Immunity and Department of Medicine; Rutgers Biomedical and Health Sciences, Institute for Infectious and Inflammatory Diseases, Rutgers University, Newark, New Jersey, United States of America; 5 Department of Pathology, University of Texas Medical Branch, Galveston, Texas, United States of America; Ulm University Medical Center, GERMANY

## Abstract

Ebolavirus (EBOV) belongs to a family of highly pathogenic viruses that cause severe hemorrhagic fever in humans. EBOV replication requires the activity of the viral polymerase complex, which includes the cofactor and Interferon antagonist VP35. We previously showed that the covalent ubiquitination of VP35 promotes virus replication by regulating interactions with the polymerase complex. In addition, VP35 can also interact non-covalently with ubiquitin (Ub); however, the function of this interaction is unknown. Here, we report that VP35 interacts with free (unanchored) K63-linked polyUb chains. Ectopic expression of Isopeptidase T (USP5), which is known to degrade unanchored polyUb chains, reduced VP35 association with Ub and correlated with diminished polymerase activity in a minigenome assay. Using computational methods, we modeled the VP35-Ub non-covalent interacting complex, identified the VP35-Ub interacting surface, and tested mutations to validate the interface. Docking simulations identified chemical compounds that can block VP35-Ub interactions leading to reduced viral polymerase activity. Treatment with the compounds reduced replication of infectious EBOV in cells and in vivo in a mouse model. In conclusion, we identified a novel role of unanchored polyUb in regulating Ebola virus polymerase function and discovered compounds that have promising anti-Ebola virus activity.

## Abstract 2

Ebola virus replication relies on the viral polymerase complex, which includes the co-factor VP35. This study reveals that unanchored polyubiquitin chains modulate polymerase function by interacting with VP35; inhibiting this interaction reduces viral replication.

## Introduction

Ebola virus disease (EVD), characterized by severe hemorrhagic fever, is caused by the highly transmissible and lethal *Orthoebolavirus*. The most pathogenic member of this family is *Zaire ebolavirus* (EBOV), which has been responsible for the highest number and most devastating outbreaks in Africa. EBOV is a non-segmented, negative-sense RNA virus from the genus *Orthoebolavirus* (family Filoviridae*)*. The viral RNA genome is encapsidated by the nucleoprotein (NP), which binds to the RNA-dependent RNA polymerase (RDRP) complex and transcription activator (VP30). The RDRP is composed of the catalytic subunit of the polymerase L and the polymerase cofactor protein VP35, which interact with the viral envelope through the VP40 and minor VP24 matrix proteins to form the virion [1–3]. In addition to VP35’s critical role as a cofactor of the viral polymerase, it has been extensively studied for its function in the inhibition of innate signaling pathways and antagonism of antiviral type-I Interferon (IFN-I) [4–15].

EBOV infection is initiated upon the virus binding to cell receptors via the viral glycoprotein (GP). EBOV enters the host cells either by pinocytosis, clathrin-mediated, or caveolin-mediated endocytosis [1,16,17]. Once the viral genome is released into the cytoplasm, transcription, and replication occur through a positive sense RNA (antigenome) and is carried out by the viral RDRP. VP35 can interact with several host proteins as well as the viral polymerase to promote virus replication and do so either via viral polymerase activity or by antagonizing immune signaling [4,6,12,14,15,17,18]. VP35 also is known to inhibit IFN-I production by targeting the pattern recognition receptor RIG-I [12]. The C-terminal region of VP35, termed the IFN-inhibitory domain (IID), inhibits IFN induction by sequestering the viral 5′-triphosphorylated dsRNA from RIG-I [8,12,19,20]. Extensive previous structural and functional work on the IID of VP35 identified 2 basic regions; a basic patch comprised of residues K222, R225, K248, and K251, involved in viral polymerase activity, and a central basic patch (R305, K309, R312, R319, R322, K339) with a major function in IFN inhibition [5,8,12,19,20]. Although the K309 residue can also affect polymerase activity [6,11]. Importantly, mutations disrupting the basic charge on R225 decreased VP35 polymerase cofactor activity, and this could be in part due to loss of interaction with NP [11].

The process of viral replication and particle release can be regulated by posttranslational modifications on different viral proteins, which additionally can alter several intracellular antiviral mechanisms [4,21–29]. Ubiquitination can enhance virus replication directly through ubiquitination of viral proteins or indirectly by modulating protein function and the innate immune response [4,21–25]. For example, in terms of direct modulation of virus replication, the HECT E3 ubiquitin (Ub) ligases SMURF2, Nedd4, ITCH, and WWP1 can promote ubiquitination of VP40 for efficient virus budding and virus-like particle (VLP) egress [23,25–30]. In addition, VP40 stability can be regulated by modification with SUMO (for Small Ub-like Modifier) [22], which can have a similar function to ubiquitination. We also recently reported that ubiquitination on VP35 is required for optimal viral transcription by promoting efficient interactions with the viral polymerase L and thus plays a critical role in viral pathogenesis [4]. VP35 is covalently ubiquitinated on lysine 309 (K309) by the host E3 ubiquitin ligase TRIM6, which is crucial for efficient viral replication [4]. TRIM6 depletion or a recombinant EBOV VP35—K309R mutant virus lacking ubiquitination on K309, results in reduced viral transcription and disrupted production of viral proteins. Moreover, a mutant of VP35 lacking ubiquitination on K309 interacts with higher affinity with the viral NP resulting in dysregulated virus assembly [6]. We also previously found that VP35 can bind Ub via non-covalent interactions; however, the precise function of these VP35-Ub interactions remains unknown [4,6]. Unanchored polyubiquitin (polyUb) chains have been shown to play a role in immune signaling [31–35] and may also play a role in virus uncoating [36]. However, to our knowledge, no role has yet been identified for unanchored Ub in viral polymerase function. Here, we show that VP35 interacts with unanchored K63-linked polyUb chains, promoting efficient viral polymerase function and EBOV replication. A VP35 R225E mutant, which was previously reported to have reduced binding with NP and reduced activity in minigenome assays [11], showed reduced binding to unanchored Ub, providing a potential explanation for the reduced polymerase activity. We identified novel antiviral compounds that block non-covalent interactions between VP35 and Ub, providing a potential novel approach to the development of antiviral strategies.

## Results

### The C-terminal IID of VP35 interacts with unanchored K63-linked polyubiquitin chains

**Fig 1:**
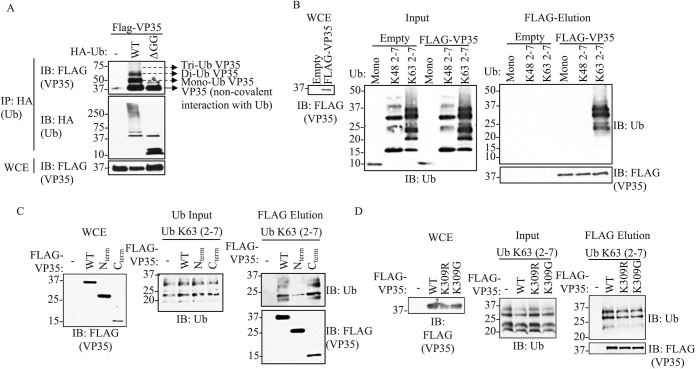
EBOV VP35 protein interacts with Ub non-covalently. (A) WCEs from HEK293T cells transfected with Flag-VP35 (VP35) and HA-Ub WT or HA-Ub ΔGG (cannot conjugate proteins) were used for HA IP under non-denaturing conditions (RIPA washes), followed by IB. (B) Purified recombinant K48 or K63 polyUb chains (mix of 2–7 Ub chains) were mixed in vitro with Flag-VP35, followed by Flag IP. Interacting proteins were eluted with Flag peptide. (C, D) Experiments performed as in (B) but using the C-terminal IID domain of VP35 (C), or the VP35 K309R or K309G mutants, which are not covalently ubiquitinated (D). * No ubiquitinated VP35, possibly phosphorylation. The data underlying the graphs shown in the figure can be found in S1 Data. EBOV, Ebolavirus; IB, immunoblot; IID, IFN-inhibitory domain; IP, immunoprecipitation; WCE, whole-cell extract; WT, wild type.

We previously reported that VP35 is covalently ubiquitinated on K309 using co-immunoprecipitation (co-IP) assays. Intriguingly, these experiments also consistently showed a non-modified fraction of VP35 that co-immunoprecipitated with Ub, suggesting a non-covalent interaction between Ub and VP35 [4]. In this new work, we postulate that VP35 interacts with unanchored or free polyUb chains and that the interaction between these Ub chains and VP35 is functionally relevant. Here, to first confirm binding between VP35 and Ub, we performed a co-IP assay in which we pulled down ectopically expressed wild type (WT) Ub or an Ub mutant lacking the C-terminal di-glycine residues (HA-Ub-ΔGG), which renders Ub unable to form covalent linkages. The terminal -GG on Ub is required for the formation of covalent conjugates of Ub with other proteins [37,38]. This approach allows testing non-covalent interactions between monomeric, non-conjugated Ub, and VP35. Consistent with our previous observation, in the presence of WT Ub, multiple migrating forms corresponding to the molecular weight of covalently ubiquitinated VP35, as well as monomeric VP35 (non-covalent interaction with Ub), were detected by immunoblot (IB). In contrast, monomeric, non-conjugated VP35, co-immunoprecipitated with HA-Ub-ΔGG (Fig 1A and IP). As expected, the HA-Ub-ΔGG runs at the predicted molecular weight of monomeric Ub (approximately 8.5 kDa) and is unable to form the characteristic smear corresponding to cellular ubiquitinated proteins (Fig 1A, whole-cell extract [WCE]). This indicates that VP35 associates non-covalently with Ub, either directly or indirectly.

To further confirm direct, non-covalent, binding between Ub and VP35 and to identify the type of polyUb chains involved in these interactions, a cell-free in vitro binding assay with purified Flag-VP35 and recombinant purified unanchored K48- or K63-Ub chains (a mix of 2 to 7 Ub chains) was conducted. We found that VP35 strongly interacts with unanchored K63- but not K48-Ub chains (Fig 1B). Furthermore, unanchored K63-polyUb chains interacted mostly with the C-terminal IID of VP35 (Fig 1C). Finally, since we previously showed that VP35 K309R or K309G mutants lose covalent ubiquitination on the K309 residue [6], we asked whether these mutants can still bind unanchored Ub. We found that these mutants interacted with unanchored Ub at similar levels compared to WT VP35 (Fig 1D), suggesting that non-covalent interactions with Ub do not require covalent ubiquitination on the K309 residue.

### Unanchored polyubiquitin chains promote Ebola virus polymerase activity

**Fig 2:**
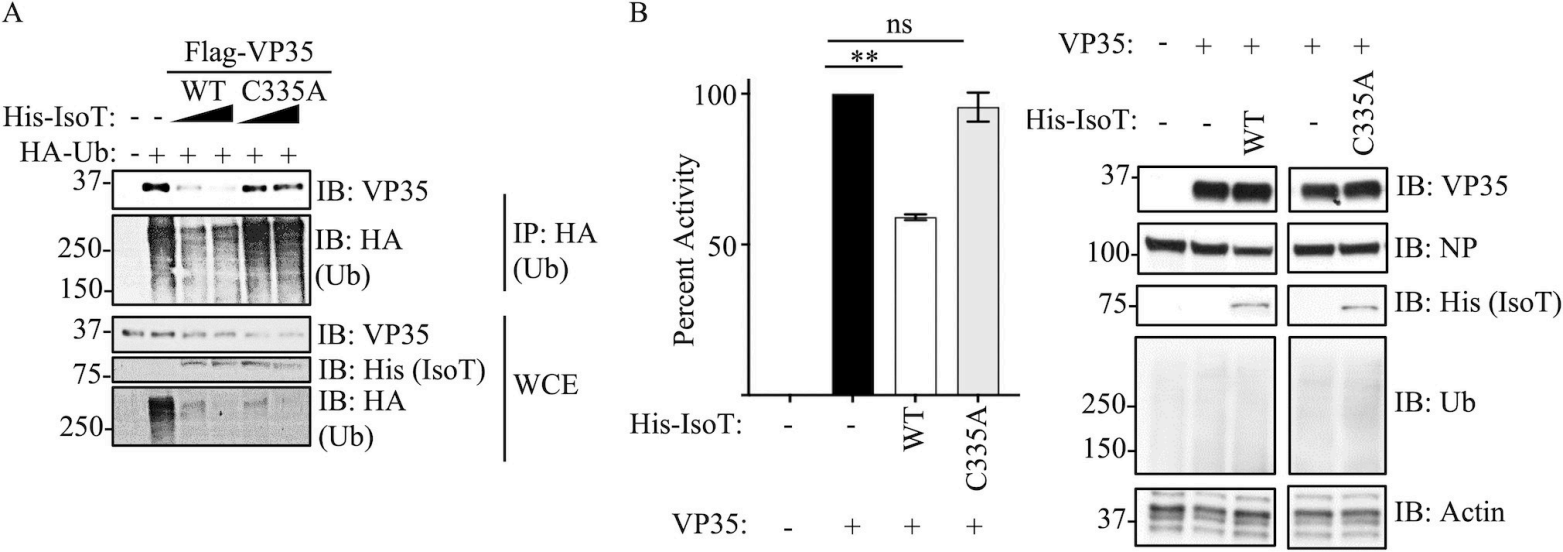
Unanchored ubiquitin interactions with VP35 promote viral polymerase activity. (A) WCE from HEK293T cells transfected with His-IsoT WT, His-IsoT C335A, VP35 WT, and HA-Ub were used for IP with anti-HA beads. (B) Polymerase minigenome assay. HEK293T cells transfected with a monocistronic firefly luciferase-expressing minigenome, including VP30, L, and REN-Luc/pRL-TK, in the presence or absence of IsoT-WT or C335A mutant. Data are expressed as mean + SEM of 3 independent assays in triplicate. Tukey’s multiple comparisons tests. ** *p* < 0.001. The percent of activity from the luciferase and renilla (Luc/ren) ratio was calculated. The data underlying the graphs shown in the figure can be found in S1 Data. IP, immunoprecipitation; WCE, whole-cell extract; WT, wild type.

Since we previously found that ubiquitination on the VP35 IID can regulate polymerase activity, we asked whether unanchored Ub would also affect this function. To test the function of unanchored Ub in relation to VP35 polymerase cofactor activity, we used the unanchored Ub-specific protease Isopeptidase T (IsoT, also called USP5), which can cleave unanchored Ub by interacting with the free di-glycine residue of Ub chains [39]. Ectopic expression of IsoT-WT cleaved polyUb chains, as observed in WCE, and correlated with reduced association of Ub with VP35 (Fig 2A). In contrast, a catalytically inactive mutant (C335A), which does not cleave unanchored Ub [39], did not affect the association between VP35 and Ub chains (Fig 2A). These results support that VP35 interacts with unanchored Ub chains. Furthermore, the effects of IsoT correlated with decreased EBOV polymerase activity evaluated in a minigenome assay (Fig 2B), suggesting that unanchored Ub may promote virus replication. However, although increasing concentration of IsoT further reduced polymerase activity, the highest concentrations do not completely abolish minigenome activity (S1 Fig), suggesting that either IsoT is unable to fully remove unanchored Ub bound to VP35, or unanchored Ub plays a partial role in promoting viral polymerase activity. The effect on the minigenome can be partially explained by enhanced interactions between VP35 and NP in the presence of unanchored K63-linked polyUb chains (S2A Fig).

### Identification of amino acid residues involved in ubiquitin–VP35 interactions

**Fig 3:**
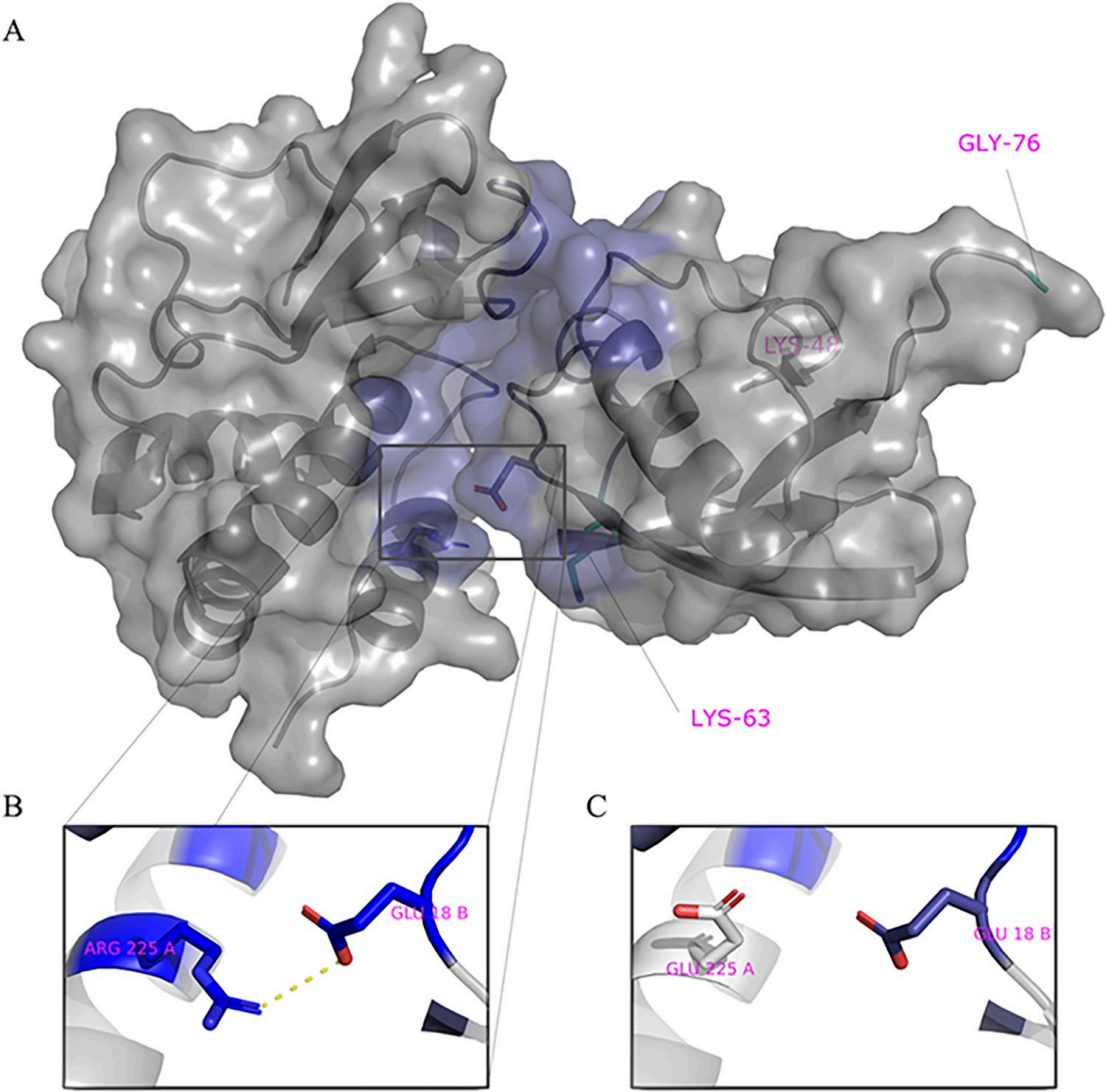
Model of VP35 interacting with Ubiquitin. (A) The complex of VP35 (PDB ID 3JKE) and Ubiquitin (PDB ID 1UBQ) modeled using a combination of protein docking and molecular dynamics simulations. Within the complex, VP35 is shown on the left and Ubiquitin on the right. The K48 and K63 Ub residues are shown in cyan on the bottom left and C-terminal on the right within Ub. (B) One of the strongest interactions contributing to the stability of the complex is ARG225-GLU18. (C) Mutation of ARG225 to GLU affects interactions. PDB, Protein Data Bank.

We employed a computational approach to identify specific amino acids in contact between VP35 and Ub. We first predicted the structure of the C-terminal IID domain of VP35 in complex with Ub, using a combination of protein docking and molecular dynamics simulations (Fig 3A).

**Fig 4:**
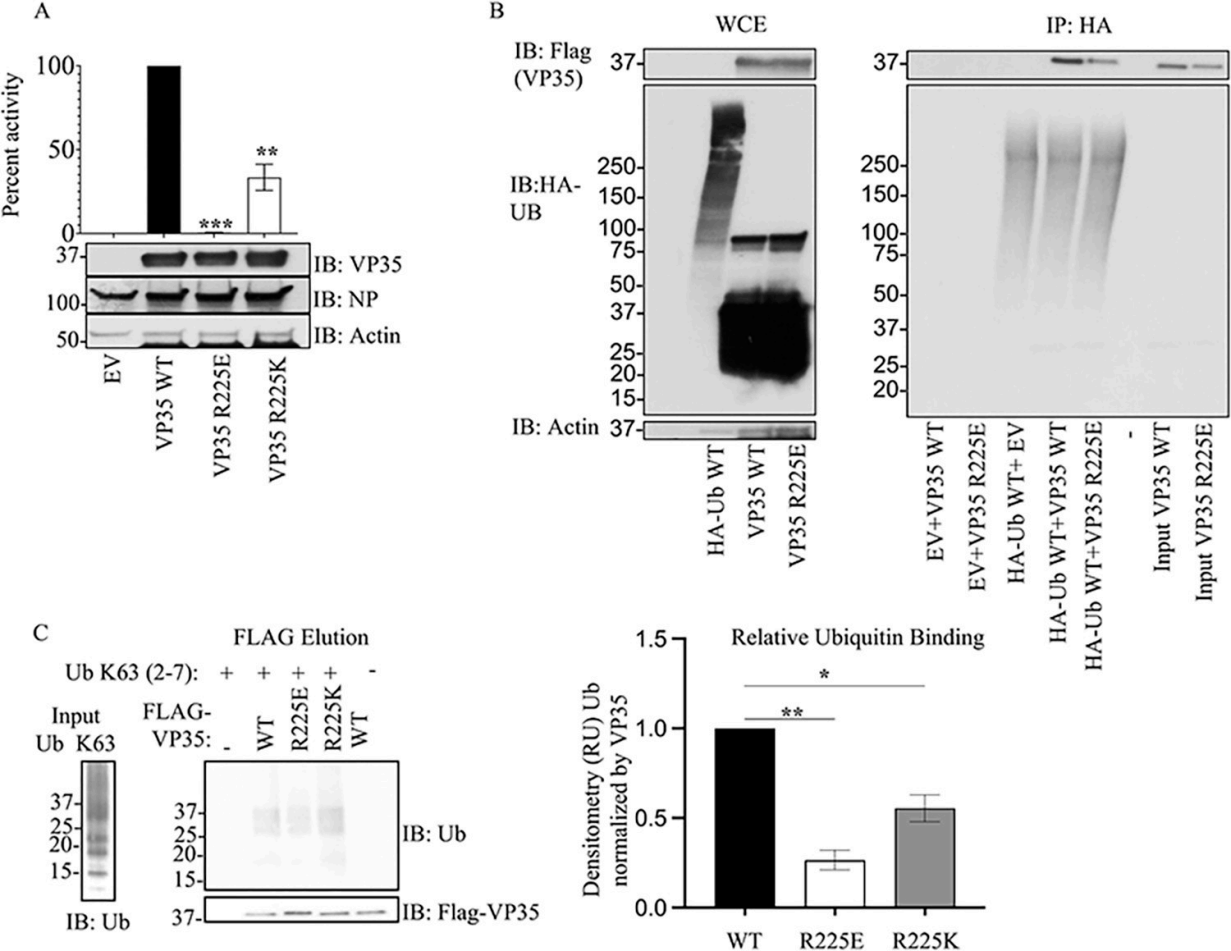
An intact R225 residue on VP35 is required for optimal interaction with Ub and viral polymerase function. (A) HEK293T cells were transfected with minigenome plasmids and VP35 WT, VP35 R225E, or VP35 R225K, followed by Luciferase assay. (B) HEK293T cells were transfected with plasmids encoding Flag-VP35 WT, VP35 R225E, or VP35 R225K. WCE were then used to isolate Flag-tagged proteins using anti-Flag beads. After washes, the beads containing VP35 were mixed with the WCE containing HA-Ub to test binding. (C) As in B, but instead of mixing with WCE, binding was performed using purified recombinant K63-linked polyUb chains, followed by Flag elution. Quantification by densitometry of 3 independent experiments is shown. The data underlying the graphs shown in the figure can be found in S1 Data. WCE, whole-cell extract; WT, wild type.

We utilized the Surfaces software to determine the top contributing interactions between VP35 and Ubiquitin. This analysis detected the R298-E24, R225-E18, R305-S57, R305-D58, and Y229-E18 as the top contributions to the VP35-Ub interaction (Fig 3A and S1 Table). We utilized gRINN to validate the Surfaces result. The gRINN analysis confirmed all 5 interactions as the top contributions (see S1 Table). Both methods suggest that R225-E18 is among the top contributors to the interaction. Analysis of all possible mutations at position R225 with Surfaces (S2 Table), suggested that the R225E mutation in VP35 would disrupt the interaction (Fig 3B and 3C). The R225E mutation was experimentally tested and has functional effects, abrogating polymerase activity in minigenome assays (Fig 4A), which is consistent with previous studies [11]. Importantly, the mutation led to a decrease in K63 polyUb binding in a cell-free in vitro co-IP assay (Fig 4B), or by mixing lysates from cells expressing VP35 and Ub (Fig 4C). In contrast, the mutation R225K, which maintains the positive charge on this residue and was predicted to not completely disrupt the interaction (S1 Table), partially rescued binding with Ub (Fig 4C). Therefore, the reduced binding of VP35-R225E and its reduced polymerase activity further supports a functional role for non-covalent interactions between VP35 and Ub in promoting viral polymerase activity and suggests that the modeled complex structure is correct.

**Fig 5:**
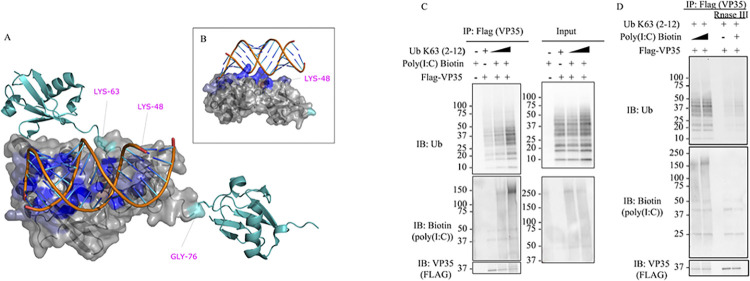
VP35-PolyUb-dsRNA predicted complex. (A) The predicted structure of the VP35-Ub complex was used as a template to superpose the structure of VP35 bound to RNA (PDB ID 3KS8). (B) PolyUb was modeled using as a template the structure of K63 Di-Ubiquitin (PDB ID 2JF5). The residues K63, K48, and G76 of the central Ub bound to VP35 are labeled in magenta and contribute favorably to RNA binding in this model. (C) In vitro competition binding assay. Increasing amounts of purified recombinant Ub K63 [2–12] were incubated with VP35 and Biotin-polyI:C, followed by IP with anti-flag beads. (D) The mixes from (C) containing VP35-Ub-PolyI:C were treated with or without Rnase III followed by IP. The data underlying the graphs shown in the figure can be found in S1 Data. IP, immunoprecipitation; PDB, Protein Data Bank.

Since our model indicates that Ub interacts with the basic patch of VP35 that modulates polymerase activity, it would not be likely that Ub affect dsRNA binding to VP35, based on previous reports [5,11]. To test this possibility, we used the core model in Fig 3A as a template to create a model of the ternary complex of VP35, dsRNA, and a tri-Ub chain of K63-linked polyubiquitin (Fig 5). Interestingly, in this model K48 PolyUb would clash with the dsRNA-binding site while the position of K63 points away from the dsRNA-binding site (Fig 5A), in agreement with the experimental data (shown in Fig 1). The model complex suggests that the central Ub bound to VP35 makes contact with RNA. Not only is K48 occluded by the RNA in the model but it in fact makes favorable interactions with the RNA (Fig 5B). Using Surfaces to identify per-residue contributions to this extended interface with RNA, we identified that K48, R54, Y59, and A46 among others favorably contribute to binding RNA. These contribute to strengthen the overall estimated binding free energy by 25% relative to that of the interface between VP35 residues and RNA but lacking the interaction with Ub. In support of this model, increasing concentrations of purified unanchored K63-linked polyUb chains did not compete with the dsRNA mimic poly(I:C) for interaction with VP35. Instead, the presence of Ub chains enhanced co-immunoprecipitation of poly(I:C) with VP35 (Fig 5C). Furthermore, treatment with RNase III, which specifically degrades dsRNA, reduced but not eliminated the interactions between VP35 and Ub (Fig 5D), further supporting a complex between Ub and VP35 that also favors interactions with dsRNA. Taken together, the interaction between Ub and RNA is likely to be functionally important and suggests that VP35-Ub interaction does not block the ability of VP35 to bind dsRNA and therefore should not affect VP35 IFN-I antagonist function.

### Identification of small-molecules that inhibit VP35–unanchored K63 Ub Interactions

**Fig 6:**
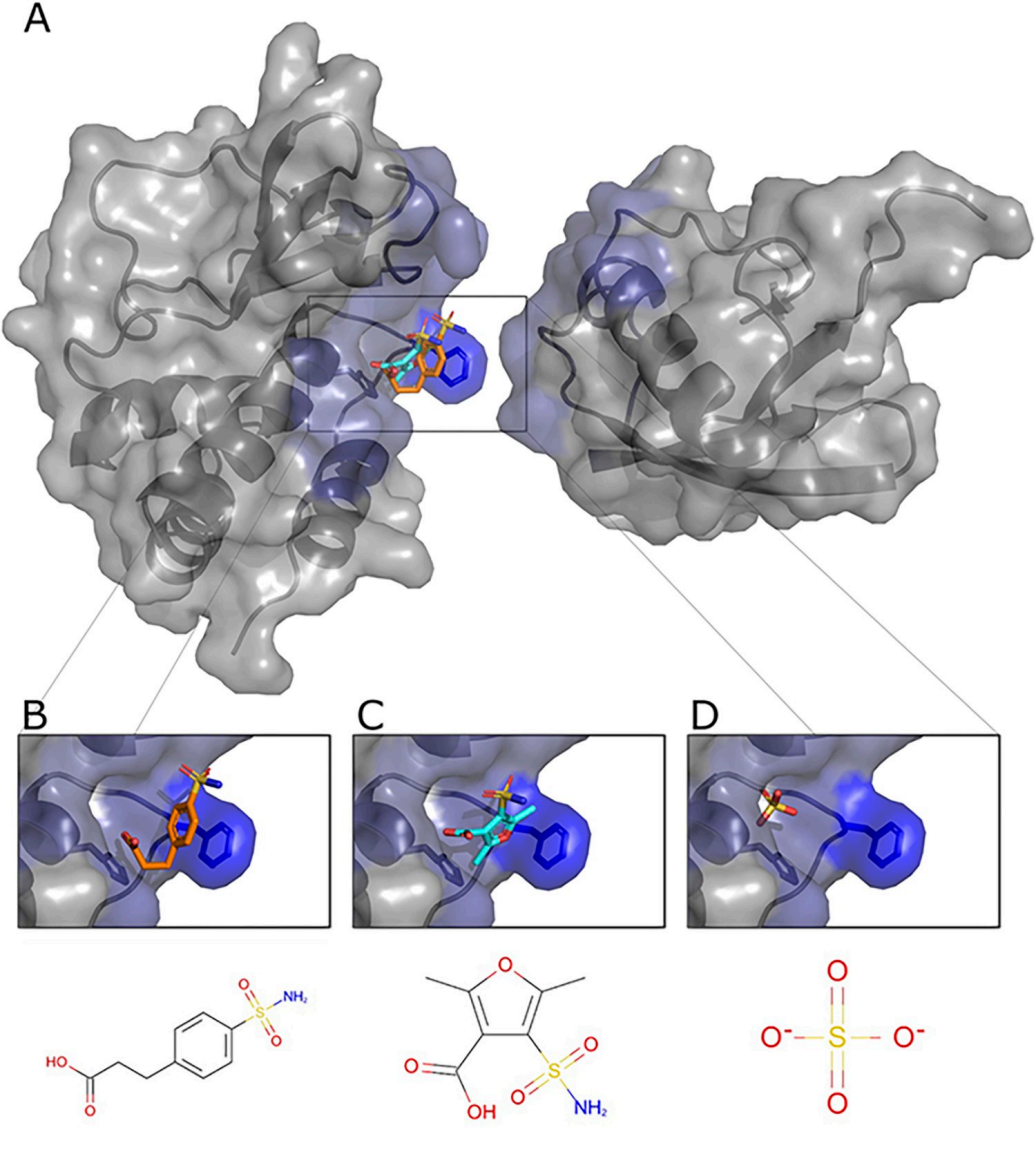
Predicted poses of small molecules disrupting the VP35-Ub interaction. (A) The cavity within the interface with Ub with predicted bound pCEBS (B) and SFC (C) in close proximity to a sulfate ion (D) observed experimentally (PDB ID 4IBG). PDB, Protein Data Bank.

To test whether the non-covalent interactions between VP35 and Ub have functional relevance, we first employed a computational approach with the objective of identifying compounds that could potentially disrupt the Ub-VP35 complex. A cavity within the putative VP35-Ub interface was used as a target to dock 36,000 small molecules with known complex structures using the small-molecule protein docking program FlexAID. Two criteria were used to detect potential binders: A combination of highly favorable docking score relative to the average of all molecules and a large level of binding-site similarities measured using the IsoMIF program between the targeted VP35 cavity and the original protein where the compound is known to bind (Fig 6A). The docking scores (CF) for the 36,000 molecules had a mean value around −100 AU. The z-score of the top 10% varied from −5.0 to −8.0. The top-scored molecules were evaluated to identify those molecules among the top 10% likely to have favorable pharmacological properties. Two molecules emerged from this analysis, pCEBS, 3-[4-(aminosulfonyl) phenyl] propanoic acid—a molecule developed to inhibit carbonic anhydrase [40], and SFC, 2,5-dimethyl-4-sulfamoyl-furan-3-carboxylic acid—a molecule developed as a Metallo-β-lactamase inhibitor [41]. The 2 candidates pCEBS and SFC, had a CF value of −321AU and −278AU, equivalent to a Z-score of −6.5 and −4.8, respectively. The binding site analysis with IsoMIF of the cavities of the crystal structures of the complexes containing SFC (PDB: 6KXO, 6KXI, and 6LBL) revealed binding site similarities of 0.25, 0.32, and 0.35 with VP35, respectively, and the cavities of the crystal structures of the complexes containing pCEBS (PDB: 2NN0 and 2NN1) showed binding site similarities of 0.24 and 0.28 to VP35, respectively. The mean binding site similarity for the top 10% of molecules in the docked dataset is 0.21. Thus, the chosen 2 molecules have a docking score considerably lower (more favorable) than the average and the binding sites known to bind these molecules are more similar to the targeted VP35 cavity than cavities of other top-scoring molecules. This suggests that important interactions responsible for binding pCEBS and SFC are also exploited in the VP35 cavity. Although independently selected, the 2 compounds share a common sulfonamide group (-S0_2_NH_2_) linked to an aromatic ring system and a carboxyl group that interacts with the same VP35 residues and both are nearly perfectly superimposed (Fig 6B and 6C). Interestingly, at least 1 X-ray structure of VP35 (PDB: 4IBG) shows a sulfate ion from the crystallization buffer bound in very close proximity to the position where the sulfonamide group from pCEBS and SFC are predicted to interact with VP35 based on the docked ligand poses (Fig 6D). The experimental observation that a sulfate ion at that position has favorable interactions with VP35 serves as indication that the docked structures with their sulfonamide groups located at that approximate position are taking advantage of interactions that were experimentally validated.

**Fig 7:**
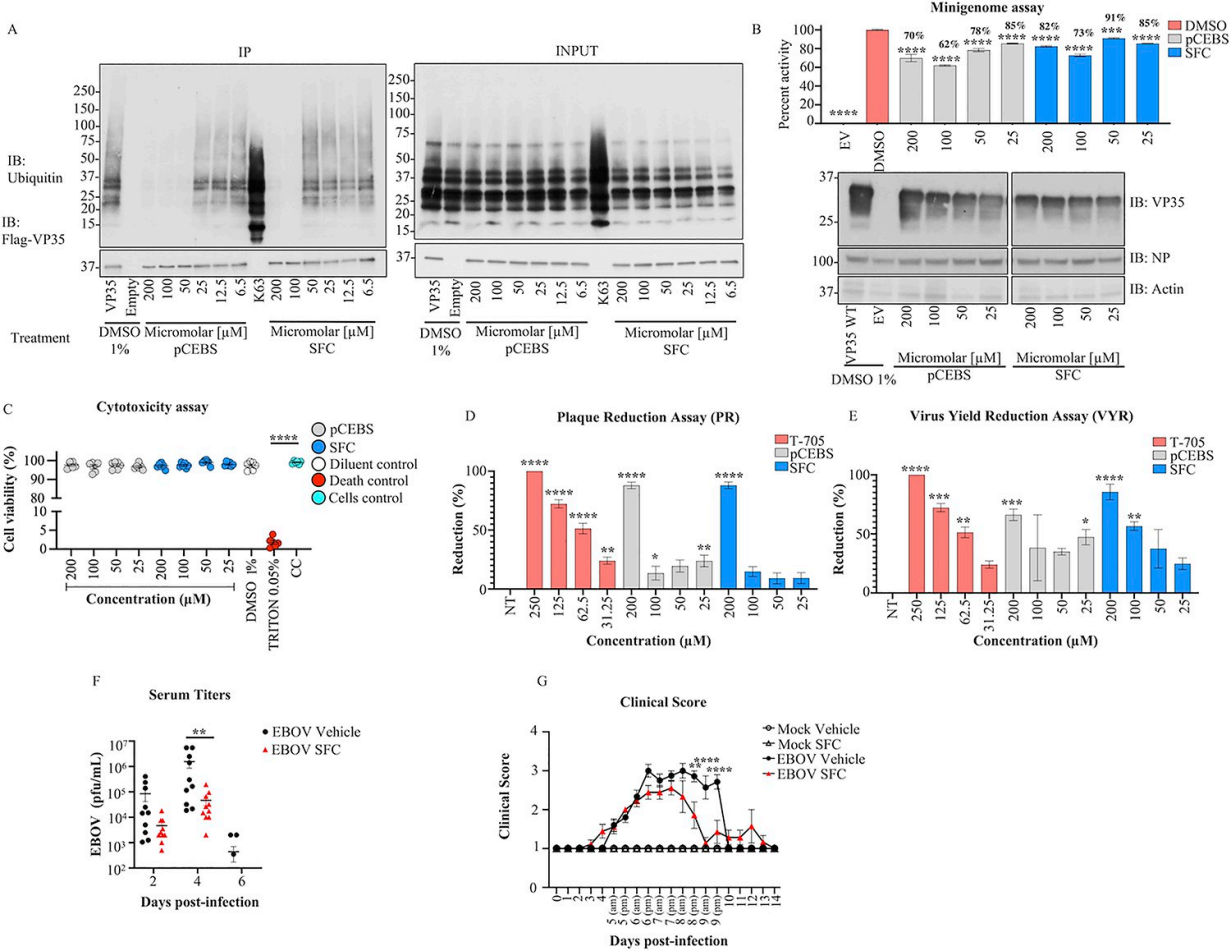
pCEBS and SFC compounds inhibit interactions between VP35 and K63-linked polyubiquitin chains and correlate with reduced viral polymerase activity and virus replication. (A) Flag-VP35 bound to anti-Flag beads were incubated for 1 h at room temperature with different concentrations of pCEBS or SFC, followed by incubation with recombinant purified unanchored K63-linked polyUb chains [2–7]. VP35-Ub complexes were eluted with Flag-peptide and analyzed by Immunoblot. (B) 293T cells were transfected with minigenome components and 4 h post-transfection cells were treated with pCEBS and SFC compounds at different concentrations, and 50 h later cells were lysed for luciferase assay. (C) Cytotoxicity test (CyQUANT MTT Cell Viability Assay Thermo Fisher) using pCEBS and SFC at different dilutions (D) PR and (E) VYR assays, the cells were infected by 1 h and after 1 h the treatment was made with pCEBS, SFB compound, or DMSO: Dimethyl sulfoxide with the overlay. The number of plaques in each set of compound dilution were converted to a percentage relative to the untreated virus control. (F, G) 6-week-old BALB/c females uninfected and treated with PBS (Mock vehicle) (*n* = 5), uninfected treated with 100 mg/kg of SFC (*n* = 5) (Mock SFC), infected intraperitoneal (IP) with 100 PFU of maEBOV and treated with either vehicle (EBOV vehicle) (*n* = 10) or SFC (EBOV SFC) (*n* = 10). (F) Viral titers in serum of infected mice at days 2, 4, and 6 post-infection. No plaques were detected in the mock groups. (G) Clinical presentation of disease scored as 1: Healthy; 2: Ruffle fur and/or Lethargic; 3: scoring 2 + hunched posture; 4: Weight loss over 20% of initial weight or scoring 3 + unable to move when stimulated, unable to access food/water, or displaying a moribund appearance. The percent of activity from the ratio of luciferase and renilla (Luc/ren) was calculated. Data are depicted as mean + SEM of the 2 independent assays in triplicate. Tukey’s multiple comparisons tests. *p* < 0.001 **, *p* < 0.0001 ***, *p* < 0.00001 ****. The data underlying the graphs shown in the figure can be found in S1 Data. EBOV, Ebolavirus; PR, plaque reduction; VYR, virus yield reduction.

Incubation with the 2 highest concentrations of either of the 2 molecules leads to a decrease in Ub-VP35 interactions detected in co-IP assays (Fig 7A) and these correlated with a decrease in luciferase activity in minigenome experiments (Fig 7B). These effects from the compounds on minigenome activity can be partially explained by reduced interactions between VP35 and NP as observed in a co-IP assay (S2B Fig). The compounds did not affect interactions between unanchored K63-linked Ub and RIG-I, which is also known to interact with free Ub [35] (S3 Fig), and served as a control for specificity. Although the inhibition did not show a perfect dose-response, possibly due to other important interactions between Ub and viral polymerase proteins, these results, at high concentrations, further suggest that Ub-VP35 non-covalent interactions may contribute to efficient EBOV polymerase function. In contrast, the compounds did not affect the ability of VP35 to antagonize IFNβ in a luciferase reporter assay (S4 Fig). The compounds showed less than 5% cytotoxicity (Fig 7C) and did not cause significant cell death (apoptosis or necrosis), by flow cytometry (S5 Fig). Importantly, both molecules lead to a decrease of infectious EBOV replication in cells, as observed in plaque reduction (PR) and virus yield reduction (VYR) assays, (Figs 7D, 7E, S6A, and S6B). The effect of pCEBS and SFC are within the same range as that observed for the nucleoside analog Favipiravir (T-705), used as a positive control for its broad-spectrum reported activity against Filoviruses [42]. However, pre-treatment with the compounds did not inhibit virus replication (S6C Fig).

To further validate experimentally direct interactions between VP35 and Ub, and VP35 and the compounds, microscale thermophoresis (MST) binding assays were performed with tagged full-length purified WT VP35 and K63-linked polyUb chains as well as with pCEBS and SFC. The MST binding experiments detected binding in all 3 instances (S7 Fig). Furthermore, MST titration curve experiments determined a K_d_ of 15 nM for Ub (S8A Fig) and estimated 375 nM for SFC (S8B Fig). It is interesting to note that the K_d_ for Ub falls well within the broad range of K_d_ observed for protein–protein interactions and can be considered as a strong interaction [43]. These results demonstrate that there are direct interactions between VP35 and Ub, as well as between VP35 and both pCEBS and SFC.

In order to add further evidence for the direct interaction between pCEBS and SFC, we modeled and evaluated computationally potential mutations that would disrupt their interactions without impairing the interface with ubiquitin and therefore not compromising the studied mechanism. We modeled 228 mutants, for all possible single substitutions in residues that constitute the binding cavity (Y229, G234, F235, G236, T237, H240, Q241, Q244, I303, P304, R305, A306). The interactions between VP35 mutants and Ub, pCEBS, or SFC (in their positions modeled to bind WTVP35) were evaluated with Surfaces [44]. Based on these results, the mutation F235H (S9 Fig) was selected as likely to not affect Ub binding, while decreasing the strength of interactions with pCEBS and SFC. We utilized the molecular docking program FlexAID [45,46] to dock the 2 molecules to the mutant structures for further evidence of weaking the interaction between the predicted mutated VP35 cavity and the 2 compounds. The FlexAID scoring function (CF) gave a more negative result for more favorable binding interactions. As FlexAID utilizes a probabilistic optimization method, we performed 5 simulations with each of the molecules. The F235H mutation decreases the CF values to −118.9 +- 2.4 AU and −128.9 +- 4.2 for pCEBS and SFC, respectively, relative to the WTVP35 values of −150.1 +- 5.3 AU and −161.5 +- 16.8 AU, respectively. Therefore, we obtain a positive ΔCF = CF_mut_-CF_wt_ of 31.2 +- 5.82 AU and 32.6 +- 17.32 AU for pCEBS and SFC, respectively. Whereas the docking score is in arbitrary units (AUs), the negative or positive sign in the ΔCF reflects an increase or decrease in favorable interactions, respectively.

To validate the model, we then generated a vector expressing the VP35 F235H mutation and tested whether the compounds are now unable to inhibit VP35 interactions with free Ub chains by coIP. This mutation was previously shown to have reduced minigenome activity [11,20]. As predicted, while the compounds reduced binding of K63-linked polyUb chains with WT VP35, the compounds did not inhibit binding of polyUb chains with VP35-F235H (S10 Fig).

Finally, we then tested whether SFC has antiviral activity in an in vivo mouse model of EBOV infection. We chose SFC because previous studies have shown that it was nontoxic in mice and has activity against carbapenem-resistant *Enterobacteriaceae* by inhibiting their metallo-ß-lactamases in vitro and in vivo [41]. Intraperitoneal administration of SFC daily for 6 days (S11 Fig) significantly reduced EBOV replication in the serum of infected mice as compared to PBS treated mice (Fig 7F). This reduced viremia in SFC treated mice also correlated with significantly less symptoms of disease such as ruffle fur, lethargy, hunched posture, or moribund appearance (Fig 7G).

Taken together, these results suggest that VP35 non-covalent interaction with Ub promotes EBOV replication by enhancing the function of VP35 as a cofactor of the polymerase. Furthermore, the identification of chemical compounds that block these VP35-Ub interactions could serve as starting point for the development of novel antivirals.

## Discussion

In this study, we demonstrated that the VP35 protein of EBOV interacts non-covalently with unanchored K63-linked polyUb chains. This interaction between Ub and VP35 promotes viral polymerase activity leading to optimal virus replication. Our findings indicate that these Ub chains play a functional role in cells and in vitro, and this is supported by the following lines of evidence: (i) identification of specific residues on VP35 in contact with Ub; (ii) mutations that change the basic amino acid on VP35 R225 reduce interactions with Ub and correlate with reduced polymerase activity; (iii) mutation on K309, which is the acceptor of covalent ubiquitination, does not affect interaction with Ub; (iv) ectopic expression of IsoT, which specifically degrades unanchored Ub, reduced viral polymerase activity and VP35-Ub binding; (v) biophysical assays demonstrate that VP35 interacts directly with Ub; and (vi) chemical compounds predicted to block VP35 interactions with Ub reduce minigenome polymerase activity and infectious EBOV replication, in cells and in vivo.

Using a pharmacological approach to block Ub-VP35 interactions, we showed that these interactions are relevant in promoting polymerase activity and replication of infectious EBOV. However, loss of Ub interaction with VP35 did not show a perfect correlation with the reduced polymerase activity and virus replication. In addition, the compounds did not show a linear dose response demonstrating the challenges of inhibiting protein–protein interfaces [47], particularly in this case where it is possible that any of the Ub units within the Ub chain have additional potential interactions, not only with RNA as modeled here for the central Ub, but also with the N-terminal region of VP35 or other viral proteins. Furthermore, it is also possible that pCEBS and SFC have additional cellular targets that affect the minigenome assays. Alternatively, Ub interactions with VP35 could affect protein oligomerization or formation of large molecular complexes and breaking these interactions with pCEBS and SFC could have larger effects on virus replication. For example, increasing amounts of VP35 result in a “bell curve” observed in minigenome assays [4,48,49] and could potentially be due to oligomerization and/or aggregation. Although we cannot rule out indirect effects of the compounds on other cellular or viral proteins at this stage, our data using VP35 mutants and small molecules that block Ub interactions with VP35 suggest that unanchored Ub plays a role, at least in part by promoting viral polymerase function.

The 2 compounds pCEBS and SFC add to the list of existing compounds that target VP35 activity. As the emergence of resistance mutations within viral proteins is common, the identification of novel compounds that likely interact with VP35 at a different binding site than previously exploited [50], contributes to the future development of novel antivirals.

VP35 has been extensively studied mostly as a major IFN-I antagonist, and its role as a cofactor of the viral polymerase is less understood. VP35 also participates in nucleocapsid packaging and efficient viral replication. Previous studies have shown that the R225 amino acid is located on a second basic patch on VP35, consisting of residues K222, R225, K248, and K251 [5]. Amino acids in this basic patch most likely do not contribute to VP35 binding to dsRNA [5], and therefore should not play a major role in IFN antagonism consistent with our computational model. In fact, the model in the presence of Ub and dsRNA suggests that the non-covalent interactions with Ub may even enhance IFN antagonism. However, the R225E mutation on VP35 reduces its activity in minigenome assays, most likely due to reduced interaction with NP, and not a loss of interaction with L [11]. Our model suggests that VP35-R225 is in contact with Ub and reduced interaction with Ub may explain the reduced minigenome activity previously observed. In fact, treatment with the compounds reduced interactions between VP35 and NP. Our studies also suggest that the compounds do not have an effect on IFN-I production.

Interestingly, while amino acids 222, 248, and 251 are conserved in all species of ebolavirus [5,51], the residue 225 in Reston virus (RESTV) and Sudan virus (SUDV) is a lysine, and in Marburg virus (MARV) is an alanine. The retention of a basic amino acid in RESTV and SUDV demonstrates some conservation in VP35 interaction with Ub, albeit at reduced levels (see S1 Table and Fig 4C). However, the R225A mutation in MARV suggests reduced Ub binding and reduced polymerase activity. Overall, this points to the possibility that ebolaviruses are undergoing evolutionary changes on VP35 via interactions with the Ub system, although it is still unclear its relationship with pathogenesis since MARV is also highly pathogenic.

We have so far been unable to identify the E3-Ub ligase responsible for the production of these unanchored polyUb chains. It seems unlikely that TRIM6 is the E3 ligase involved in this process because we have shown TRIM6 can covalently modify VP35 [4] and can also synthesize unanchored K48-linked polyUb chains [34]. In addition, the mechanisms by which covalent ubiquitination on K309 and binding of unanchored Ub seem distinct since K309 is located in the central basic patch and its ubiquitination promotes binding to L leading to regulation of viral transcription [6], while binding of unanchored Ub to the first basic patch of VP35 is most likely regulating binding with NP [11].

The function of unanchored Ub continues to be controversial. It has been proposed to regulate different immune pathways and inflammatory responses by the host [52]. For example, we found that unanchored K48-linked polyUb regulates the antiviral response by promoting IKKε activation to stimulate IFN-I responses [34], and K63-linked polyUb chains can promote RIG-I activation [31,33,53–55], and TAK1 activation [35,56,57]. On the other hand, viruses have been shown to utilize both unanchored Ub and covalent ubiquitination to replicate. In relation to unanchored Ub, Influenza viruses contain unanchored Ub in infectious virions, which can enhance virus replication by promoting uncoating via histone deacetylase 6 (HDAC6) [58,59], and other viruses may use similar mechanisms [60]. We have also detected Ub chains in sucrose-purified infectious EBOV, which could be covalently attached to VP35 or other viral proteins (S12 Fig); however, Ub corresponding to low molecular weight chains suggesting some unanchored Ub may be present (S12A Fig). Currently, it is unclear if these Ub chains would act at the level of virus entry or would prime VP35 to initiate its polymerase cofactor activity early during infection. This further supports a role for Ub in promoting early stages of virus replication.

Despite the existence of approved treatments for patients infected with EBOV, which include monoclonal antibodies mAb114 and REGN-EB3 [61,62], outbreaks of EBOV in Africa continue to present a public health threat. Therefore, there is still a need for the identification of accessible treatments. In conclusion, we have used a computational approach combined with the identification of chemical compounds that block non-covalent VP35-Ub interactions to study functional outcomes. This approach exemplifies how computational docking can contribute to the study of host–virus interactions and predict potential therapeutic approaches.

## Materials and methods

### Ethics statement

Mice experiments were carried out in accordance with Institutional Animal Care and Use Committee (IACUC) of the University of Texas Medical Branch at Galveston, with protocol approval # 2203018, and were performed under animal biosafety level 4 (ABSL4). No human participants or donors were used in these studies.

### Plasmids and reagents

The VP35 constructs (VP35 R225E and R225K mutations) in the pCAGGS backbone were kindly provided by Dr. Basler (Icahn School of Medicine at Mount Sinai, NY) and were previously described [11]. The mutations on VP35 K309R and K309G were previously described in [6]. The IsoT plasmid was described in [34]. The mutant plasmid sequences were confirmed using Sanger sequencing (UTMB Molecular Genomics). The other plasmids including Renilla luciferase, pCAGGS empty vector, and minigenome components (EBOV L, EBOV NP, EBOV VP30, T7 polymerase, and EBOV minigenome firefly luciferase) have been previously described [4,6].

### Cells and viruses

Cells were obtained from the American Type Culture Collection (Manassas, VA). Vero cells (ATCC CCL81), and Vero E6 were used for infection studies, and HEK293T cells (ATCC CRL-3216) were used for transfection. Cells were maintained in 1X Dulbecco’s Modified Eagle’s Medium (DMEM) (Gibco by Life Technologies, USA), supplemented with 5% fetal bovine serum (FBS) low in endotoxins and heat-inactivated (Life Technologies, Rockville, Maryland, USA) and incubated at 37°C, with 5% CO_2_. WT Ebola virus (EBOV) and mouse-adapted EBOV (maEBOV) strain Zaire were propagated in Vero E6 cells. Virus titer was determined by plaque assay using Vero-CLL81 cells [6]. All experiments performed with infectious EBOV were carried out in the Robert E. Shope and Galveston National Laboratory Biological Safely Level 4 laboratories at The University of Texas Medical Branch at Galveston.

### Modeling the non-covalent interaction between VP35 and ubiquitin

Protein–protein docking of Ub on VP35 was done using ClusPro 2.0 [63] and ZDOCK [64] web servers with default parameters, using Protein Data Bank (PDB) [65] entry IDs 1ubq and 3jke, respectively. The top 10 poses from ZDOCK were kept for minimization. ClusPro divides the results into 4 categories: balanced, electrostatics-favored, hydrophobics-favored, and Van der Walls + Electrostatics favored. The top 5 poses from the balanced category and the top 3 from each other were kept (14 total from ClusPro).

These 24 poses were then submitted to minimization, equilibration and short 100 pico second (ps) production molecular dynamics trajectories using GROMACS [66] version 2016.3, with parameters as described in the lysozyme tutorial from [67]. The conformations and energies from the trajectories were saved every 2 ps. 3 replicate trajectories were computed for every of the 24 poses. The average total potential energies from these short runs were used to rank the different poses, and the top 4 poses were selected this way. These 4 poses were then simulated for 3 replicates of 1ns trajectories, using the same parameters as before.

The resulting trajectories were analyzed using the gRINN software [68] to find pairs of residues of low interaction energy at the protein–protein interface. We used default parameters with a stride of 10 (50 frames from each replicated 1 nano second (ns) trajectory were analyzed). We were aiming to find pairs common to all docking poses and all frames, but surprisingly there were none. Instead, we chose to analyze only the 3 replicate trajectories from the lowest energy pose, according to the potential energy computed from the MD simulations (pose 4 from ZDOCK). This allowed us to rank all VP35-Ub interface pairwise residue interactions in decreasing order of relative contribution to the predicted binding free energy (ΔG).

We employed Surfaces [44], a software to quantify and visualize interactions within and between proteins and ligands. With Surfaces, we were able to evaluate the change in ΔG expected from each of the 19 mutations on VP35 for the top-ranked interacting position.

### Modeling interactions between VP35, Ubiquitin, and dsRNA

We modeled the complex involving VP35, Ubiquitin, and dsRNA using our modeled complex of VP35 and Ub as a template upon which we superimposed experimental structures of VP35 in complex with dsRNA (PDB: 3KS8) and K63-linked Ubiquitin dimer (PDB: 2JF5). In order to model a chain of 3 K63-linked Ub monomers, we used twice the 2JF5 structure, and superimposed the first chain of the dimer once and the second chain onto the Ub once. We utilized the Yasara energy minimization web server [69] to resolve any steric clashes in the ternary complex between the VP35-Ub complex and dsRNA. The minimized structure was further validated for the absence of steric clashes using the dedicated function provided by Surfaces scripts [44].

### Identifying potential protein–protein interaction disruptors of VP35-Ubiquitin through ultra-massive virtual screening

We used GetCleft, a C implementation of the Surfnet algorithm [70] to identify the top 5 largest cavities of VP35 using the structure generated by molecular dynamics. From these, we selected the cavities in contact with residues in the interface with Ub with the rationale that a molecule occupying such a cavity may disrupt binding to Ub. For the docking experiments, we selected molecules from the Chemical Component Dictionary [71] representing all ligands in complex with a protein present in the PDB. We selected all compounds composed of more than 4 non-hydrogen atoms of which 2 are carbons, for a total of 36,000 compounds. We utilized the ligand protein docking software FlexAID [45] to perform 10 docking simulations (500 generations and 500 chromosomes, or 250,000 pose evaluations) for each of the 36,000 molecules and ranked the molecules based on the mean docking score value (CF score). We selected the top 10% molecules for a second round of 10 docking simulations of 1,000 generations and 1,000 chromosomes (1 × 10^6^ evaluations). We proceeded to compare the level of binding site similarities between the binding site defined by each of the 20 top-ranked molecules and their known binding site as seen in the PDB. For that, we utilized the IsoMIF [72] method for the detection of molecular interaction field similarities. The 2 compounds ultimately selected for experimental testing were molecules with high values of binding site similarities and high docking scores but also favorable pharmacological properties.

### Plaque reduction (PR), virus yield reduction (VYR), and cytotoxicity assays

The antiviral activity of pCEBS and SFC against EBOV were determined in Vero CCL-81 cells as described in [73].

### Transfections and immunoprecipitations

HEK293T cells were plated in 6-well plates (400,000 cells/well) in DMEM 1X supplemented with 5% FBS for 24 h, followed transfection with the specific plasmid (Flag-VP35 WT, Flag-VP35 R225E, Flag-VP35 R225K, Flag-VP35 K309R, Flag-VP35 K309G, Flag-VP35 N-terminus, Flag-VP35 C-terminus, HA-Ub WT, HA-Ub ΔGG, His-IsoT WT, His-IsoT C335A, and pCAGGS empty vector), using TransIT-LT1 (Mirus) following the manufacturer’s recommendations, and 30 h after transfection, cells were lysed in RIPA buffer containing complete protease inhibitor (Roche), n-ethylmaleimide (NEM), and iodoacetamide (IA) (RIPA complete). Lysates were cleared at 25,200 xg for 20 min at 4°C, and 10% of the clarified lysate was added to 2× Laemmli sample buffer (BioRad) with 5% ß-mercaptoethanol and boiled at 95°C for 10 min to generate WCEs. The remaining clarified lysate was mixed with 10 μl of anti-Flag-Agarose or anti-HA-Agarose beads (Sigma) and incubated at 4°C overnight on a rotating platform. Later, the beads were washed 7 times with RIPA buffer (SDS 0.1% (v/v), Deoxycholic acid sodium salt 0.5% (w/v), Tris (pH 8.0) 50 mM, NaCl 150 mM, NP-40 1%) with IA and NEM, after the 7 RIPA washes. If elution was not necessary, the beads were mixed with 50 μl 2× buffer Laemmli and boiled to 95°C for 10 min. For elution, 7 RIPA washes, an additional wash made using peptide elution buffer (10 mM Tris (pH 7.4) and 150 mM NaCl in nuclease-free water (NF H2O)) without peptide were done. The protein was then eluted in 15 μl of peptide elution buffer 3 times using Flag peptide or HA peptide (Sigma) 300 μg/ml. Finally, 4× buffer Laemmli was added to each elution and boiled to 95°C for 10 min.

To test Ubiquitin interactions, an additional wash using NT2 buffer (Tris 7.4 50 mM, NaCl 150 mM, MgCl 1 mM, NP-40 0.05% (v/v)) was done, and the beads were kept with 200 μl of NT2 buffer and added K63 polyUb chains (2 to 7 chains) (UBPBio) and incubated at 4°C overnight on a rotating platform, and 10% of these was added to 2× Laemmli sample buffer (BioRad) with 5% ß-mercaptoethanol and boiled at 95°C for 10 min to generate the inputs. The beads were washed 7 times with NT2 buffer and elution was made as described previously.

To test Ubiquitin interactions with VP35 and small compounds, immunoprecipitations were done following a similar protocol to test Ubiquitin interactions. Briefly, an additional wash using NT2 buffer was performed and the beads were incubated with 200 μl of NT2 buffer and treated with 3-[4-(aminosulfonyl) phenyl]propanoic acid (pCEBS) (Enamine US) or 2,5-Dimethyl-4-sulfamoyl furan-3-carboxylic acid (SFC) (Enamine US) pCEBS and SFC were diluted to final concentrations of 200, 100, 50, 25, and 12 μm in 1% dimethyl sulfoxide (DMSO). The beads were incubated at room temperature (RT) for 1 h on a rotating rocker and were incubated with K63 polyUb chains (2 to 7 chains) (UBPBio) at 4°C overnight on a rotating platform; 10% of these were added to 2× Laemmli sample buffer (BioRad) with 5% ß-mercaptoethanol and boiled at 95°C for 10 min to generate the inputs. Beads were washed 7 times with NT2 buffer, and elution was done as mentioned previously.

### Minigenome assay

The monocistronic minigenome construct expressing the firefly luciferase gene [74] was kindly provided by Dr. Bukreyev (UTMB). The plasmids pCEZ-NP, pCEZ-VP35, pCEZ-VP30, pCEZ-L, and pC-T7 were previously described [75]. 293T cells were plated (50,000 cells/well) onto 24-well plates in 5% FBS 1× DMEM for 24 h, and co-transfected with the following plasmids: EBOV minigenome (125 ng), pCEZ-VP30 (31.25 ng), pCEZ-NP (62.5 ng), pCEZ-L (500 ng), pC-T7 polymerase (125 ng), 100 ng of empty vector (pCAGGS) or pCAGGS-VP35 (WT, R225E, or R225K), and REN-Luc/pRL-TK plasmid (20 ng; Promega) expressing Renilla luciferase used as an internal control to normalize transfection efficiency. To test the compounds, 4 h post-transfection, treatments with pCEBS or SFC to 200, 100, 50, 25, and 12 μm in 1% DMSO were added, and cells were incubated at 37°C, 5% CO2 for 50 h. After, the cells were lysed to measure the luciferase signal using the Dual-Luciferase Reporter Assay System (Promega) with a Cytation 5 reader (Biotek). A portion of the lysate was boiled at 95°C for 10 min in 2× Laemmli buffer to evaluate protein expression via immunoblot.

### IFNβ luciferase promoter assays

293T cells were plated in a 96-well plate (20,000 cells/well) in 10% FBS DMEM for 16 h prior to transfection. For the dsRNA-induction experiment, after 24 h of VP35 plasmid transfection (100 ng), and treated for 24 h with 200 μm of pCEBS, SFC, or DMSO as vehicle. HMW poly(I:C) (3.125 μg/ml) was transfected with Lipofectamine 2000 (Invitrogen). The cells were lysed at 16 h after poly(I:C) transfection to measure luciferase. For both experiments, 30% of lysates were collected and boiled in 4× Laemmli sample buffer (Bio-Rad).

### Biotin-poly(I:C) competition binding assay

Biotin-labeled HMW poly(I:C), 500 ng, (InvivoGen) was allowed to bind streptavidin-agarose beads (Sigma) in NT2 buffer overnight at 4°C on a rocking platform and washed 7 times in NT2 buffer to remove any unbound poly(I:C). FLAG-peptide purified FLAG-VP35 was incubated with the biotin-poly(I:C) coated beads in 200 μl NT2 buffer for 4 h at 4°C on a rocking platform. Increasing amounts of purified recombinant Ub K63 [2–12] were incubated with VP35 and Biotin-poly(I:C), followed by IP with anti-flag beads. Then, the mixes containing VP35-Ub-poly(I:C) were treated with or without Rnase III followed by IP. After 7 washes in NT2 buffer, the beads were boiled at 95°C in 2× Laemmli sample buffer for 10 min.

### Western blot

Protein samples were run on 4% to 15% or 7.5% Criterion-TGX Precast Gels (Bio-Rad). The proteins were transferred onto a methanol-activated Immun-Blot PVDF membrane (Bio-Rad), and the membrane was blocked in 5% Carnation powdered skim milk (Nestle) in 1× TBS-T (20 mM Tris, 150 mM NaCl) for 1 h. Primary antibodies were prepared in 3% bovine serum albumin 1× TBS-T with 0.02% sodium azide to the appropriate dilution: anti-Flag and anti-HA (Sigma) 1:2,000, anti-VP35 (6C5 Kerafast) 1:1,000, anti-NP (provided by Dr. Basler, Mount Sinai), anti-ubiquitin (Enzo) 1:1,000, anti-β-actin (Abcam) 1:2,000, anti-His (Sigma) 1:2,000, and anti-β-tubulin (Sigma) 1:1,000. The next day, the blot was washed 3 times every 5 min using 1× TBS-T before incubation with HRP-conjugated goat-anti-rabbit (GE Healthcare) or goat-anti-mouse (GE health care) or Streptavidin (HRP) (Abcam) for 1 h. The blot was washed in 1× TBS-T and developed using Pierce ECL Western Blotting Substrate (Thermo Fisher) or SuperSignal West Femto Maximum Sensitivity Substrate (Thermo Scientific). For blot quantifications, the area under the curve (AUC) was measured for each band of interest using ImageJ.

### Microscale thermophoresis (MST)

#### Labeling

Protein labeling was carried out according to the protocol of the labeling kit RED-NHS 2nd Generation (Nanotemper cat. # MO-L011). Briefly, 90 μl of 10 μm protein solution was mixed with 10 μl of 300 μm dye solution in the provided NHS labeling buffer, yielding 100 μl of dye-protein solution with an approx. 3-fold excess of dye. The labeling reaction was carried out for 30 min at RT in the dark. A gravity flow column included in the labeling kit was used to remove excess dye. The sample was eluted in MST buffer (50 mM Tris-HCl (pH 7.8), 150 mM NaCl, 10 mM MgCl2, 0.05% Tween 20), flash-frozen in liquid nitrogen and stored at −80°C.

#### Preparation of reaction mixtures for MST experiment

For the binding check, VP35 and K63-Ub [2–12] were diluted to a concentration of 20 nM and 2 μm, respectively, in MST buffer (assay buffer). Stock solutions of 8 μm SFC and pCEBS in 1% dimethyl sulfoxide (DMSO) were diluted to a concentration of 0.4 mM in MST buffer. For evaluation of the binding affinity to SFC, 10 μl of a 40 nM stock solution of VP35 was mixed with 10 μl of a serial dilution of SFC starting at 800 μm, while making sure that the final DMSO concentration remained constant throughout the dilution series. The samples were incubated for 15 min at RT in the dark.

#### MST binding experiment

Samples were centrifuged at 14,000 g for 10 min at 4°C, then loaded into NanoTemper Standard Capillaries (cat# MO-K002). MST analysis was performed using a NanoTemper Monolith NT.115 instrument at LED settings of 100% excitation power and medium MST power, at RT. The system was running MO.Control software v1.5.3, and data were analyzed at default MST-on times using MO.Affinity Analysis software v2.2.7.

### In vivo EBOV infection

Mice experiments were carried out in accordance with Institutional Animal Care and Use Committee (IACUC) and were performed under animal biosafety level 4 (ABSL4) conditions at the University of Texas Medical Branch at Galveston. 6-week-old BALB/c female mice (Envigo) were infected via the intraperitoneal (IP) route with 100 PFU of ma-EBOV. Treatment with either vehicle (PBS) or 100 mg/kg of SFC was performed via the IP route immediately after viral challenge, 3 h post-infection and then everyday post-infection for 6 consecutive days. Health check and body weights were recorded daily. The following clinical scoring system was used: (1): Healthy; (2): Ruffled fur or Lethargic; (3): scoring 2 plus hunched posture; (4): Weight loss over 20% of initial weight or scoring 3 plus unable to move when stimulated, unable to access food/water, or displaying a moribund appearance. Score of 4 required immediate euthanasia. At 2, 4, and 6 days post-infection, retro-orbital bleeds were collected and viremia determined via plaque assay.

### Statistical analysis

Three independent assays were carried out in triplicate for a single-factor analysis of variance to determine if differences exist among the groups: treated, positive controls, negative controls, and infection. A Shapiro–Wilk test was performed to determine normality in the data. Multiple comparison tests were performed to determine among which groups statistically significant differences are occurring compared to the infection control using Tukey’s multiple comparison test. These tests were run through the GraphPad Prism v.9 statistical package (San Diego, California, USA). All tests were considered statistically significant when *p* < 0.05 with 95% CI.

## Supporting information

S1 Data Underlying numerical data for Figs 2B, 4A, 4C, 7B, 7C, 7D, 7E, 7F, 7G, S1A, S1B, S1C, S4, S5A, S5C, S6A, S6B, S7A, S7B, S7C, S8A, and S8B. (XLSX)

S1 Raw Images Original images supporting all western blot results reported in Figs 1A, 1B, 1C, 1D, 2A, 2B, 4A, 4B, 4C, 5C, 5D, 7A, 7B, S1A, S1B, S1C, S2A, S2B, S3, S4, S10, S12A, and S12B. The experimental samples, loading order, and molecular weight markers are indicated.
(PDF)

S1 Table Comparison of contribution to binding energy of gRINN and Surfaces predictions in kcal/mol of individual interactions within the VP35-Ub complex interface. (PDF)

S2 Table Predicted contributions of different residues modeled in position 225 to the binding energy in kcal/mol overall or specifically with GLU18. (PDF)

S1 Fig Increasing concentrations of IsoT in minigenome assay partially affects polymerase activity. (A–C) HEK293T cells were transfected with minigenome components and (A) including 12.5, 25, and 50 ng of IsoT WT. (B, C) Including 50, 100, and 200 ng of IsoT WT or catalytic impaired mutant IsoT 335A, 50 h later cells were lysed for luciferase assay and western blot analysis. The data underlying the graphs shown in the figure can be found in S1 Data.
(PDF)

S2 Fig Unanchored K63-linked Ub chains enhance VP35 –NP interactions. (A) The addition of unanchored K63-linked polyUb chains enhances WT VP35 interactions with NP. Lysates from HEK293T cells expressing WT-VP35 were mixed with lysate from cells expressing NP, in the presence or absence of added purified unanchored K63-linked polyUb chains [2–12], followed by coIP with anti-Flag beads. (B) **Treatment with pCEBS or SFC in a minigenome assay reduces interactions between VP35 and NP in the co-immunoprecipitation assay**. HEK293T cells were transfected with minigenome components and 4 h post-transfection cells were treated with 200 μm of pCEBS and SFC; 50 h later cells were lysed, and immunoprecipitation assay was performed using FLAG beads.
(PDF)

S3 Fig RIG-I interactions with unanchored Ub are not affected by the compounds. HEK293T cells were transfected with FLAG-VP35 or FLAG-RIG-I. After lysis, FLAG immunoprecipitations were performed to isolate RIG-I and VP35, followed by incubation with purified K63-linked polyUb chains [2–12], in the presence of 200 μm or pCEBS or SFC, or DMSO as control.
(PDF)

S4 Fig pCEBS and SFC do not alter VP35 IFNβ antagonist activity. (A) IFN-β promoter assay HEK293T cells were transfected with 100 ng of VP35 WT and treated for 24 h with 200 μm of pCEBS, SFC, or DMSO as vehicle. Then, cells were transfected with 3.125 μg/ml of HMW Poly I:C for 16 h. (B) Western blot analysis of (A). Data are depicted as mean + SEM. Two-Way ANOVA Tukey’s multiple comparisons tests. The data underlying the graphs shown in the figure can be found in S1 Data.
(PDF)

S5 Fig pCEBS and SFC do not affect cell viability. (A) Cell viability from Vero CCL81 treated with DMSO, pCEBS, or SFC 200 μm for 10 days, metabolic activity was measured using MTT assay (Thermo Fisher). (B, C) Flow cytometry analysis of Vero CCL81 treated with DMSO, pCEBS, or SFC 200 μm for 1 or 10 days, cells were then stained using ENZO GFP-Certified apoptosis/necrosis detection kit acquired in an LSR Fortessa (BD) and analyzed using flowJo. The data underlying the graphs shown in the figure can be found in S1 Data.
(PDF)

S6 Fig Pre-treatment and post-treatment using compounds. (A, B) Ebola titers obtained from virus yield reduction assay expressed in Log10 by pfu/ml. (B) Ebola titers obtained from Plaque reduction assay expressed in Log10 by pfu/ml, shown in Fig 7D and 7E. Data are depicted as mean + SEM. One-Way ANOVA Tukey’s multiple comparisons test. (C) Pre-treatment with pCEBS and SFC do not affect EBOV replication. Fluorescent microscopy of Vero CCL81 cells untreated or treated with 200 μm of pCEBS or SFC for 1 h, compounds were removed, and cells were infected with EBOV-GFP MOI 0.01. The data underlying the graphs shown in the figure can be found in S1 Data.
(PDF)

S7 Fig Microscale thermophoresis (MST) binding assay of VP35 with Ubiquitin and Compounds. Tagged WT VP35 in complex different interactors to determine if there are shifts normalized fluorescence (Fnorm) (A) K63-linked Ubiquitin (a mixture of *n* = 3–12), (B) pCEBS, and (C) SFC. The data underlying the graphs shown in the figure can be found in S1 Data.
(PDF)

S8 Fig Microscale thermophoresis (MST) titration. (A) Microscale thermophoresis (MST) titration curve for the VP35 Ubiquitin interaction. Normalized fluorescence (Fnorm) for Tagged WT VP35 in complex with different concentrations of Ubiquitin were measured by MST. The estimated K_d_ is 15 nM. (B) Microscale thermophoresis (MST) titration curve for the VP35 SFC interaction. Normalized fluorescence (Fnorm) for Tagged WT VP35 in complex with different concentrations of SFC were measured by MST. The estimated K_d_ is 375 nM. The data underlying the graphs shown in the figure can be found in S1 Data.
(PDF)

S9 Fig Interactions between wild type (WT) VP35 protein and the F235H mutant with ubiquitin and compounds. Interactions between wild type (WT) VP35 protein (in gray) and the F235H mutant with ubiquitin (in green) on the left column, pCEBS (in orange, middle column), and SFC (in cyan, right column), with the dash lines representing pairwise atomic interactions that change by more than 0.1 kcal/mol between WT and F235 as predicted by Surfaces. The colors of individual dash lines, as well as the colors of entire residues, are shown in a scale of blue to white in which blue represents more favorable interactions/residues with stronger net interactions and white represents less significant interactions/residues with net interactions closer to null. Compared to WT, we see that the F235H mutation in VP35 strengthens the interaction with residues PRO19, SER57, ASN60, ILE61, and most of all with GLN62 of Ubiquitin (on the right in the 2 left-most panels). Compared to WT, the F235H mutations do not abolish interactions between this residue (H235) for either of the compounds. In both cases, however, the small molecules are predicted to have weaker interactions with residue H235 compared to F235. The overall predicted DG of binding (in kcal/mol) for each complex is displayed in each panel. The difference between the bottom and top DG values for each interaction gives a predicted DDG of −1.57 kcal/mol for the interaction with Ubiquitin, suggesting a strengthening of this interaction, whereas for pCEBS and SFC we obtain positive DDG values of 0.23 kcal/mol and 0.74 kcal/mol, respectively, suggesting that both molecules have a weaker interaction with VP35 in the F235H mutant.
(PDF)

S10 Fig pCEBS and SFC inhibit WT -VP35 but not VP35 F235H unanchored Ub binding in coIP. Lysates from HEK293T cells expressing VP35 WT or VP35 F235H were incubated with anti-FLAG beads to isolate VP35. After washes, purified recombinant unanchored K63-linked polyUb chains [2–12] were incubated with the beads containing VP35 as described. After washes, Ub bound to VP35 was detected by Immunoblot (IB).
(PDF)

S11 Fig In vivo treatment with SFC during EBOV infection. (A) Scheme of the in vivo mouse experiment.
(PDF)

S12 Fig Infectious EBOV particles contain free Ubiquitin and a proportion is associated with VP35. Recombinant EBOV WT or VP35 K309R mutant virus was purified by sucrose gradient. Purification was performed as described in van Tol and colleagues [6]. In short, Supernatants from a T75 flasks of VeroE6 cells infected at an MOI of 0.01 PFU/cell were collected at 144 hpi for sucrose-gradient purification. The 15 ml of supernatant was first clarified to remove cellular debris before loading onto a 25% sucrose cushion. The virus was loaded onto a 20%–60% sucrose gradient and then pelleted. The pellet was resuspended in STE buffer and an aliquot was used directly for immunoblot shown in (A), as input virus. The rest of sample was used for immunoprecipitation (IP), using an anti-VP35 antibody or an IgG control, shown in (B). To obtain evidence that VP35 associates with free ubiquitin in the virion, the same amount of sample used for IP was boiled before proceeding to IP, to denature all proteins (B, right side). Ubiquitin running at high molecular weight (over 100 kDa) is dissociated from VP35, suggesting some long free ubiquitin chains are associated with VP35 non-covalently. Little difference is observed between WT and K309R viruses, suggesting that most of the ubiquitin packaged in the virion is not covalently attached on the K309 site of VP35. Aliquots of the purified virus were used for titration to demonstrate infectivity, shown in reference [6]. In panel A, based on the molecular weight of 1 unit of Ubiquitin (approximately 8.5 kDa), the purified virions contain at least free Ub and Ub chains in the form of mono-Ub, di-Ub, tri-Ub, and tetra-Ub. Longer forms of polyubiquitin chains can be detected but cannot be differentiated between free ubiquitin or covalently modified VP35, or other potential viral proteins covalently ubiquitinated. VP30, VP40, and NP are shown to demonstrate the presence of EBOV particles after the sucrose purification.
(PDF)

## Abbreviations

AU: arbitrary unit
AUC: area under the curve
co-IP: co-immunoprecipitation
DMEM: Dulbecco’s Modified Eagle’s Medium
EBOV: Ebolavirus
EVD: Ebola virus disease
FBS: fetal bovine serum
GP: glycoprotein
IB: immunoblot
IID: IFN-inhibitory domain
MARV: Marburg virus
MST: microscale thermophoresis
NP: nucleoprotein
PDB: Protein Data Bank
PR: plaque reduction
RDRP: RNA-dependent RNA polymerase
RESTV: Reston virus
RT: room temperature
SUDV: Sudan virus
VLP: virus-like particle
VYR: virus yield reduction
WCE: whole-cell extract
WT: wild type
