# Supplementary material for: Ebola virus VP35 interacts non-covalently with ubiquitin chains to promote viral replication
Source: PLoS Biol. 2024 Feb 29;22(2):e3002544. doi: 10.1371/journal.pbio.3002544 (PMC10942258; doi:10.1371/journal.pbio.3002544)

Figure 1 Uncropped images of western blots.

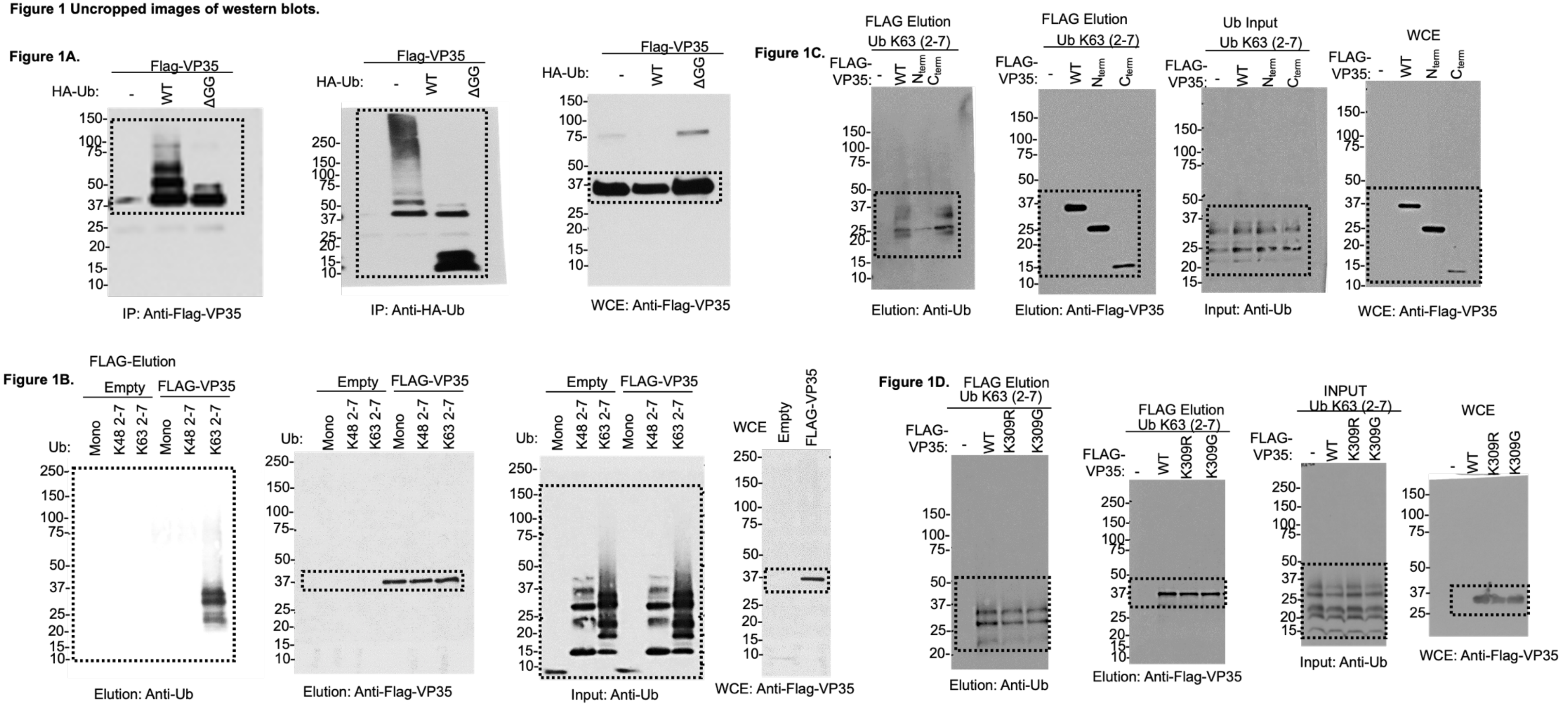

Figure 2. Uncropped images of western blots.

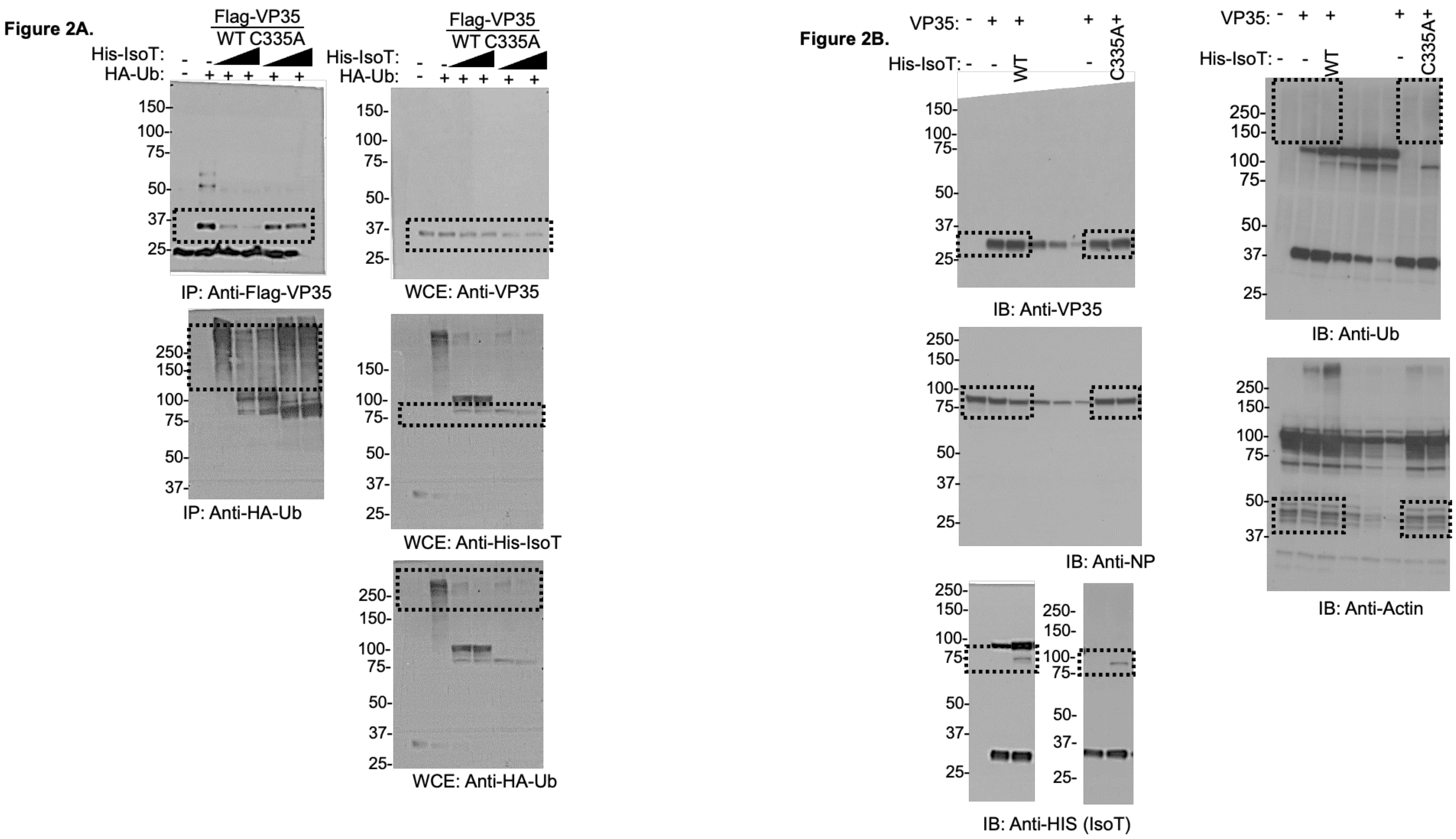

Figure 4. Uncropped images of western blots.

Figure 4A.

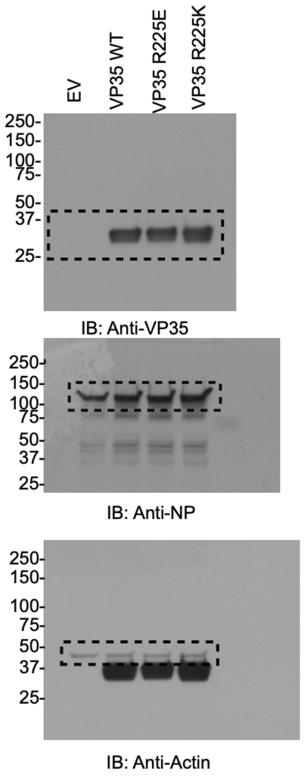

Figure 4B.

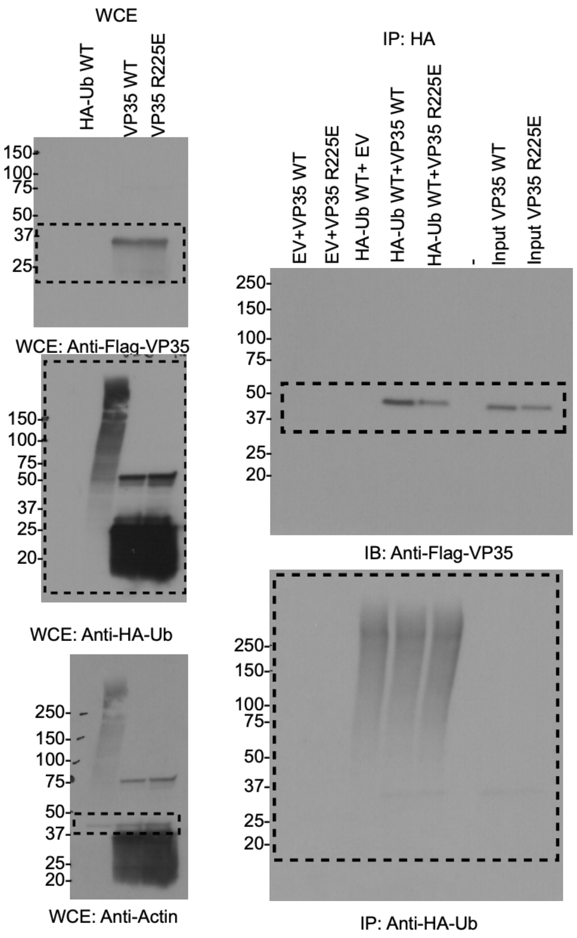

Figure 4C.

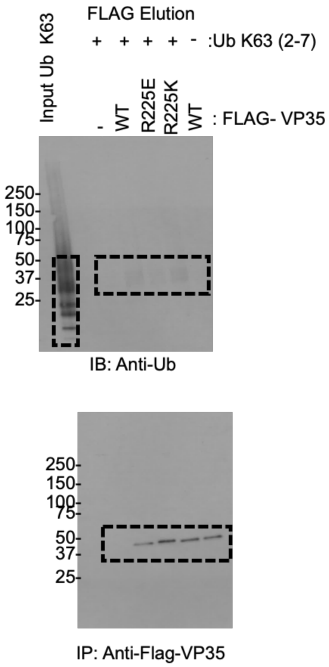

Figure 5. Uncropped images of western blots.

Figure 5C.

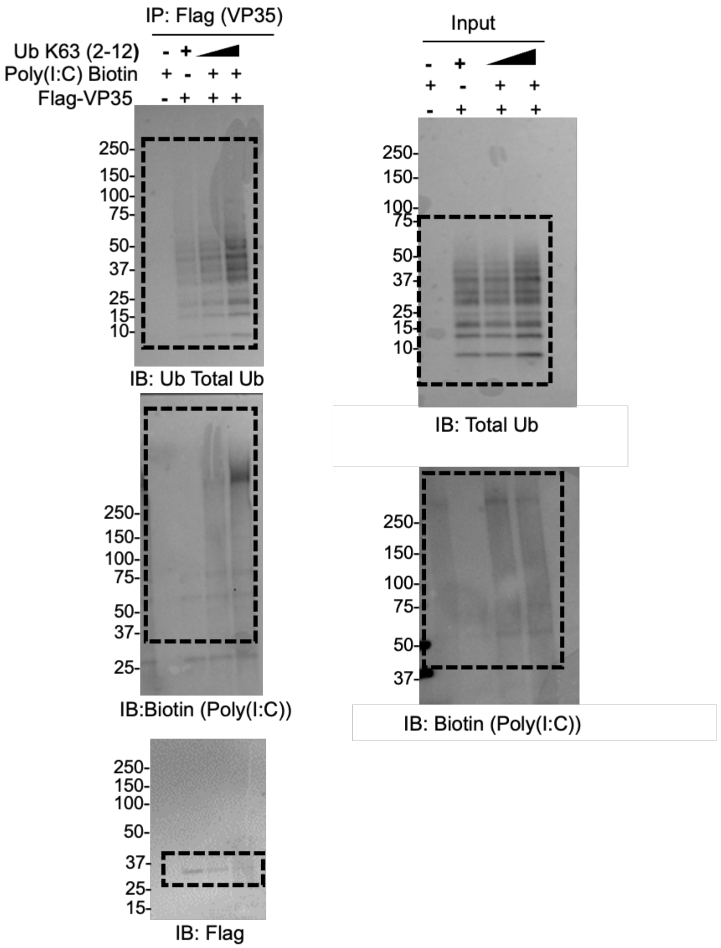

Figure 5D.

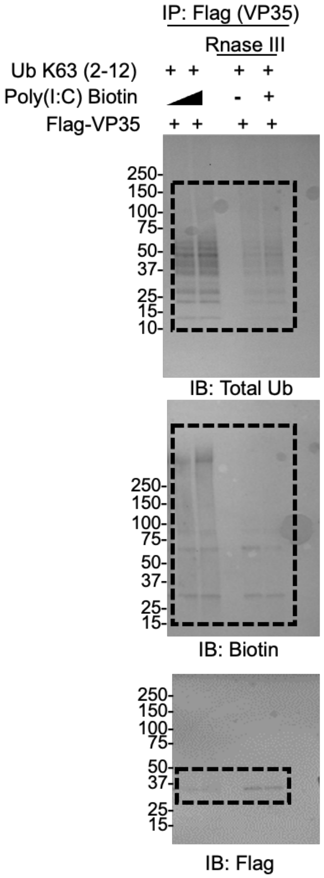

Figure 7. Uncropped images of western blots.

Figure 7A.

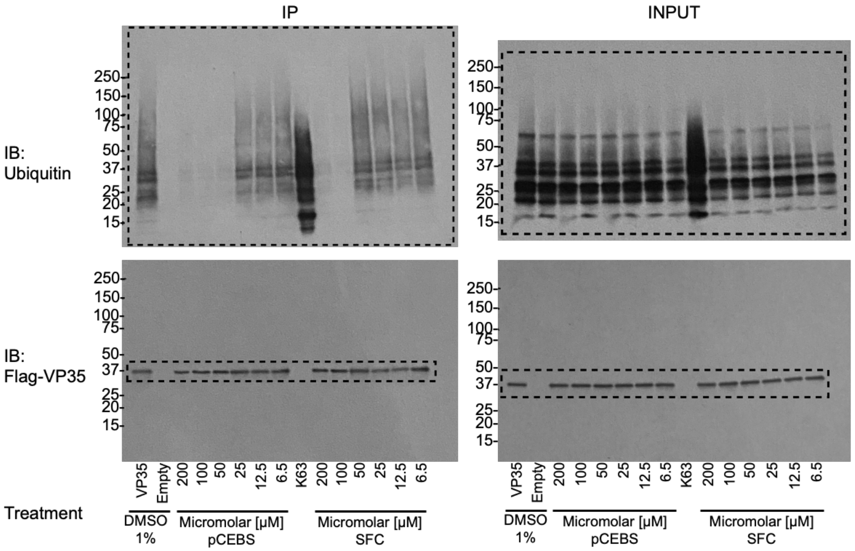

Figure7B.

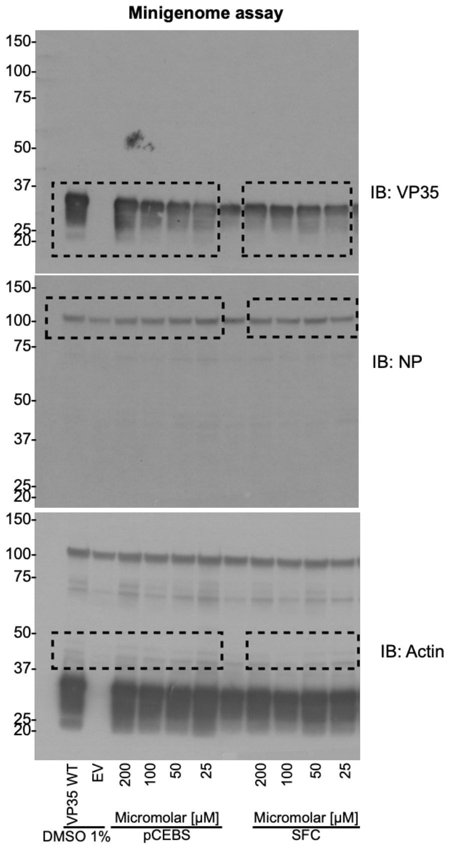

Supplementary Figure 1. Uncropped images of western blots.

Figure S1A. Minigenome experiment 1.

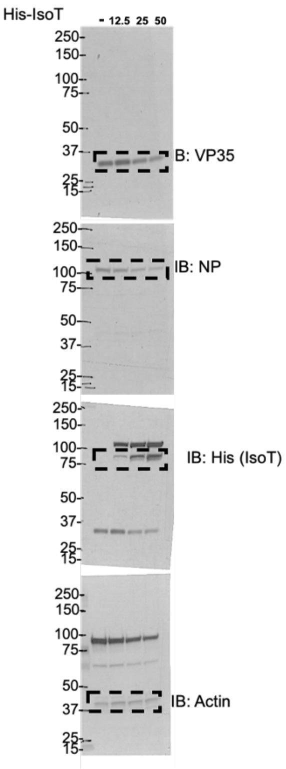

Figure S1B. Minigenome experiment 2.

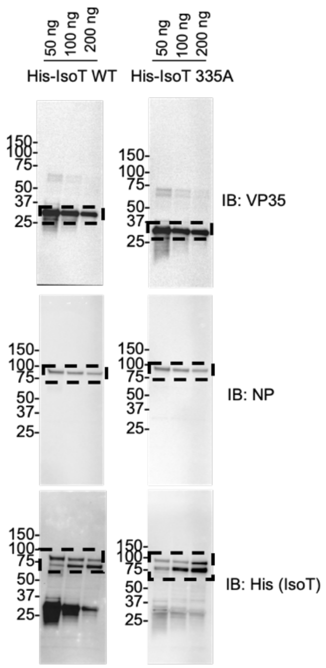

Figure S1C. Minigenome experiment 3.

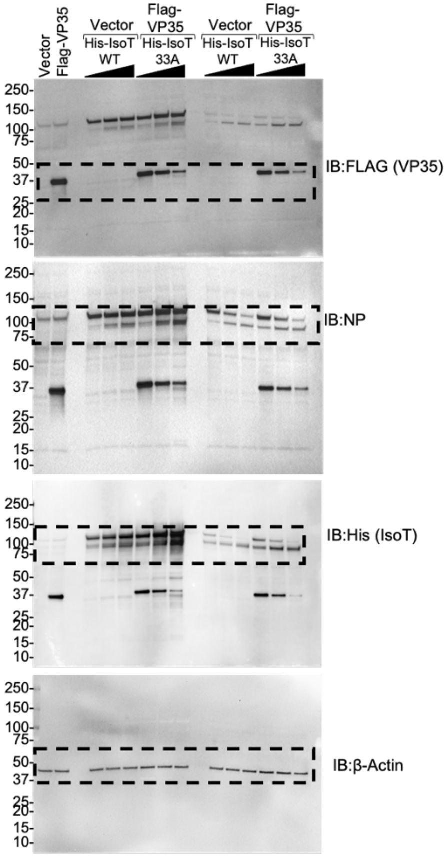

Supplementary Figure 2. Uncropped images of western blots.

Figure S2A

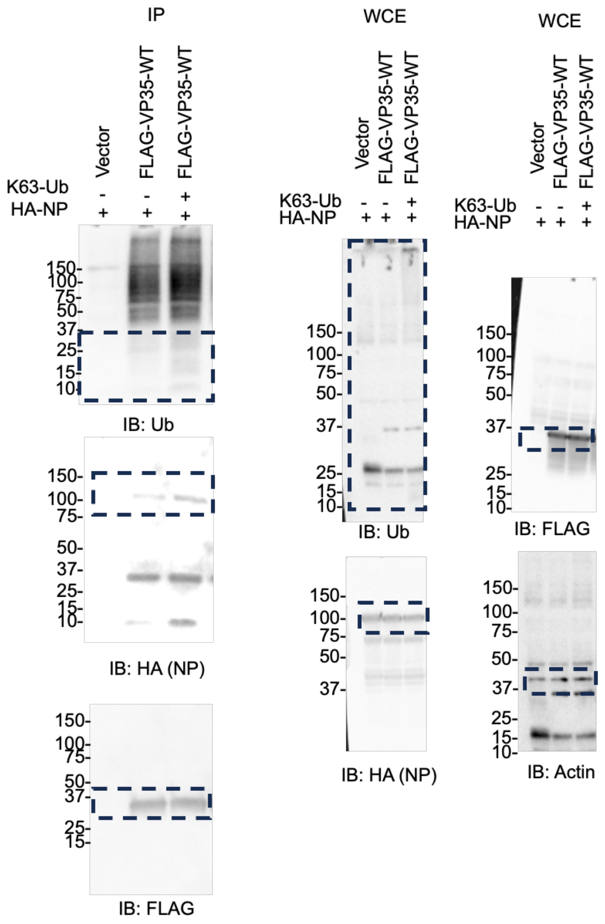

Figure S2B

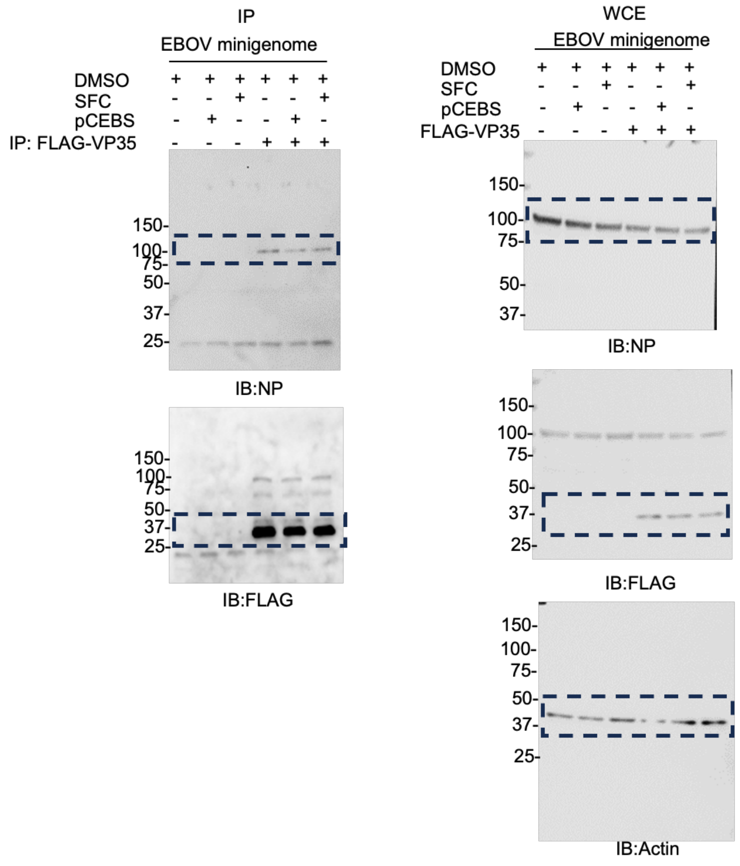

Supplementary Figure 3. Uncropped images of western blots.

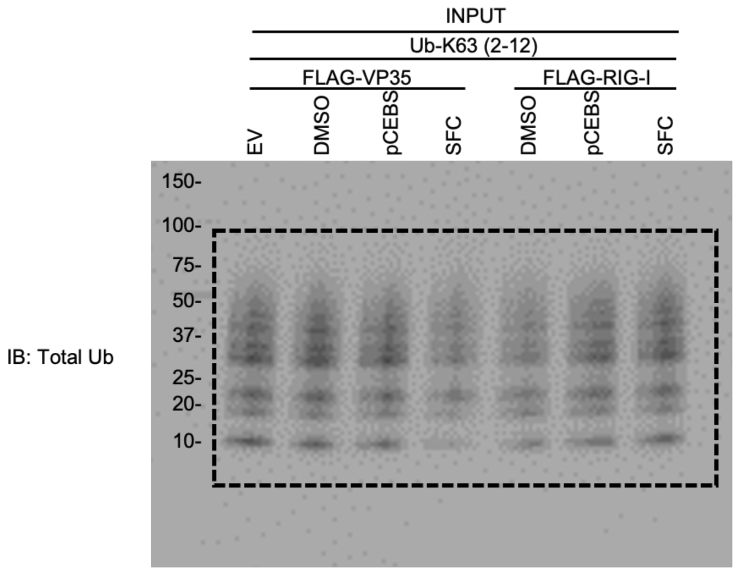

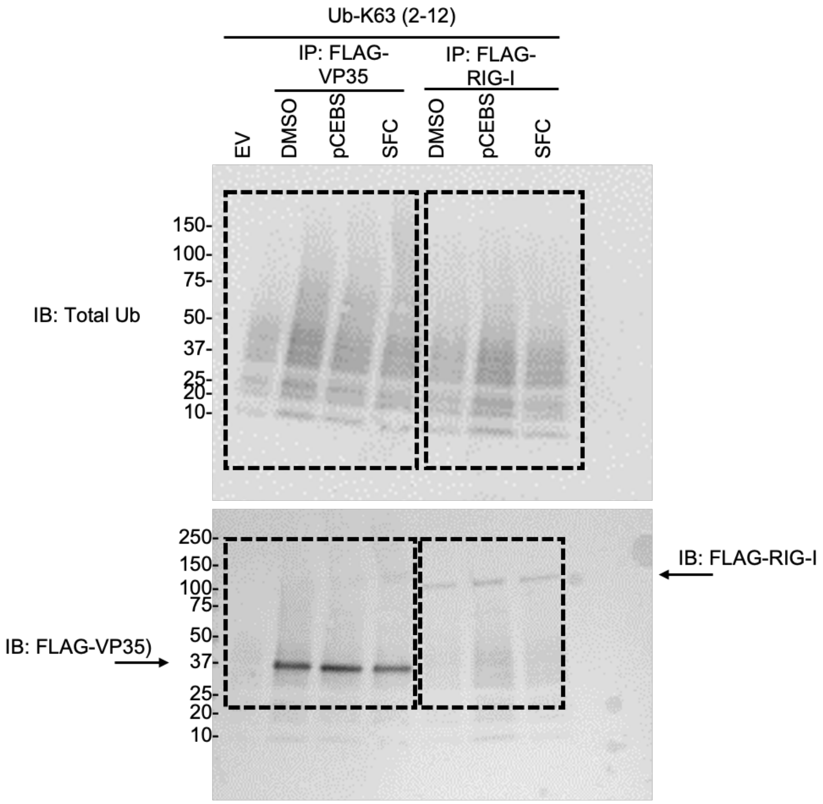

Supplementary Figure 4. Uncropped images of western blots.

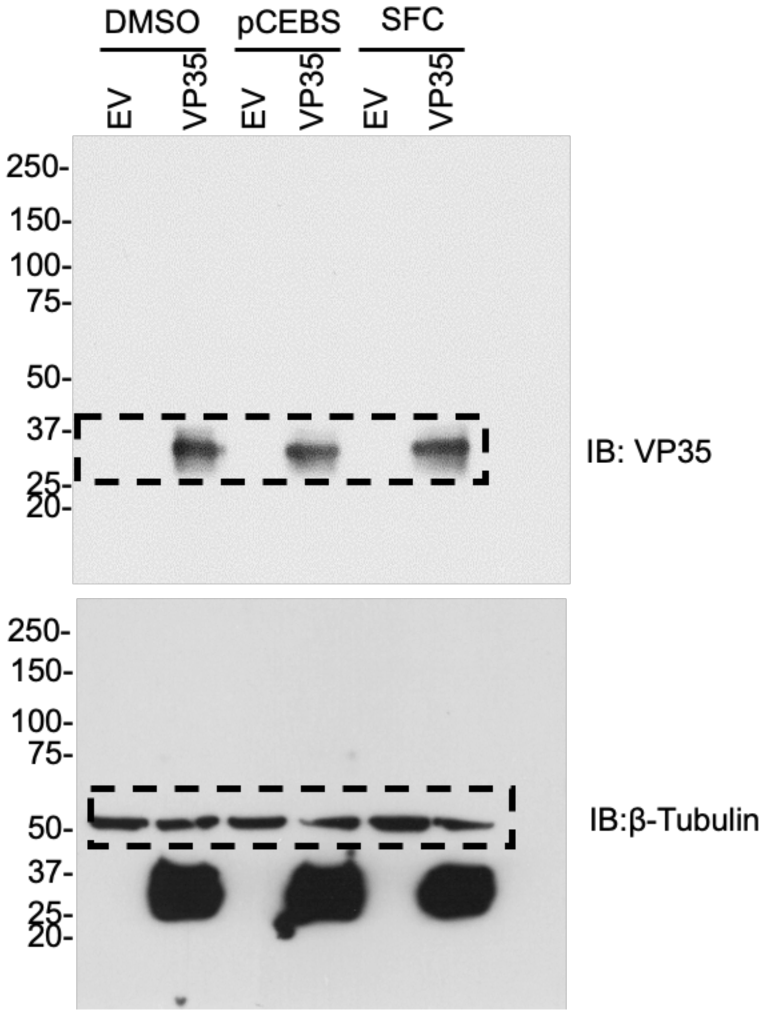

Supplementary Figure 10. Uncropped images of western blots.

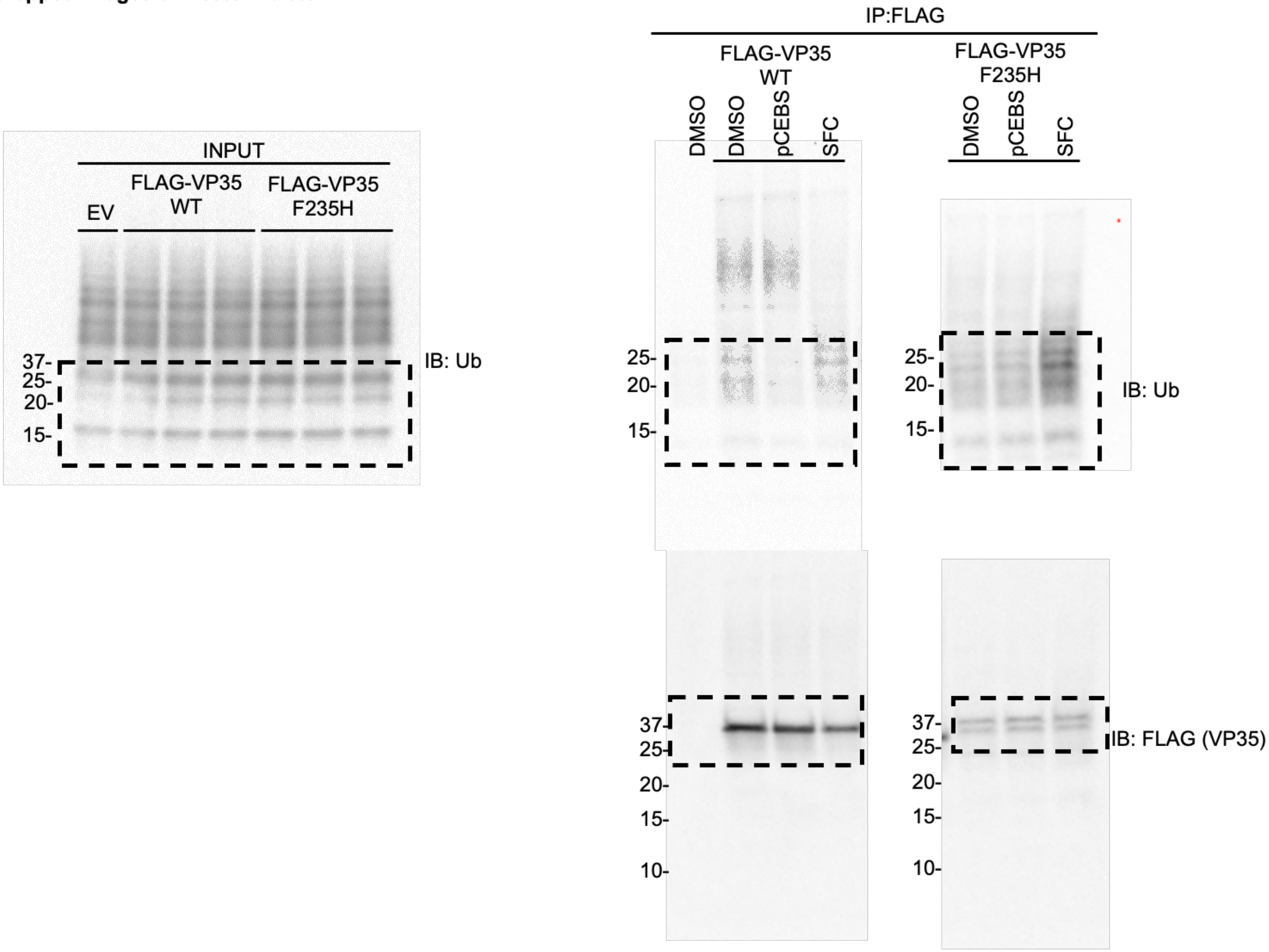

Supplementary Figure 12. Uncropped images of western blots.

Figure S12A

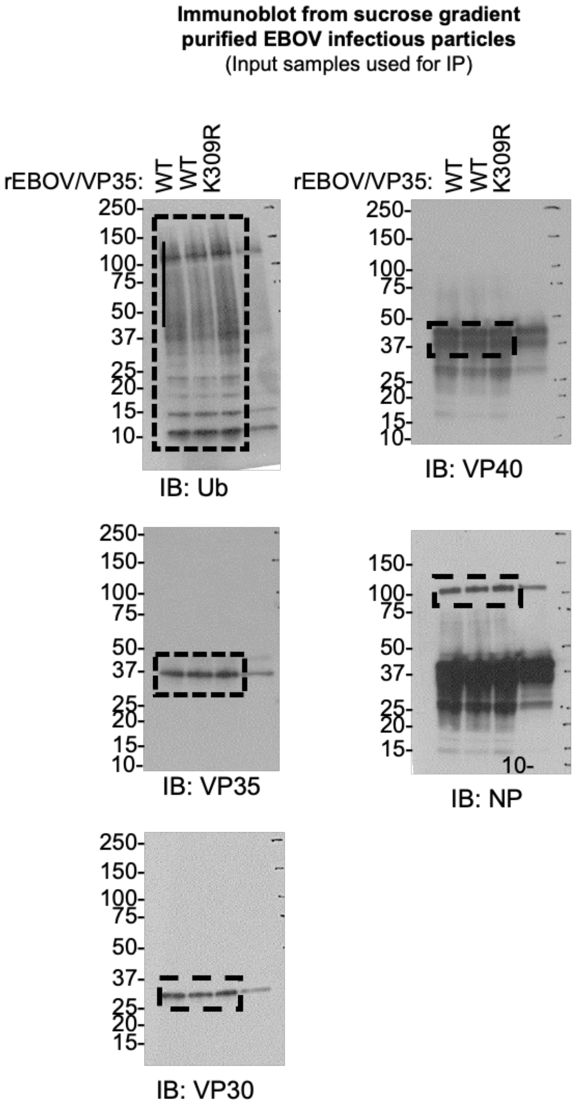

Figure S12B

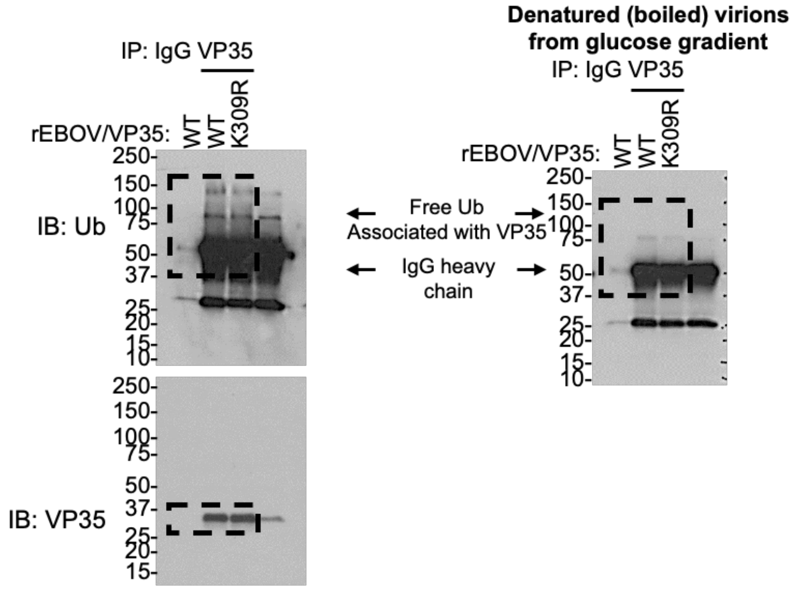

Supplement: S1 Raw Images — The experimental samples, loading order, and molecular weight markers are indicated. (PDF) [file pbio.3002544.s002.pdf]
