# Supplementary material for: Ebola virus VP35 interacts non-covalently with ubiquitin chains to promote viral replication
Source: PLoS Biol. 2024 Feb 29;22(2):e3002544. doi: 10.1371/journal.pbio.3002544 (PMC10942258; doi:10.1371/journal.pbio.3002544)
Supplement: S1 Table — (PDF) [file pbio.3002544.s003.pdf]

S1 Table. Comparison of contribution to binding energy of gRINN and Surfaces predictions in kcal/mol of individual interactions within the VP35-Ub complex interface.

VP35	Ub	gRINN	Surfaces
ARG225	GLU18	-14.81	-1.11
LYS222	GLU16	-14.00	0.00
ARG305	ASP58	-10.60	-0.75
ARG298	GLU24	-10.43	-1.85
ASP230	LYS63	-9.00	0.00
TYR229	GLU18	-4.45	-0.59
GLN244	GLU18	-1.99	-0.01
PRO304	ASP58	-1.65	0.00
ARG305	SER57	-1.63	-0.99
LYS222	GLU18	-1.60	0.00
