# Supplementary material for: Ebola virus VP35 interacts non-covalently with ubiquitin chains to promote viral replication
Source: PLoS Biol. 2024 Feb 29;22(2):e3002544. doi: 10.1371/journal.pbio.3002544 (PMC10942258; doi:10.1371/journal.pbio.3002544)
Supplement: S2 Table — (PDF) [file pbio.3002544.s004.pdf]

**S2 Table**. Predicted contributions of different residues modeled in position 225 to the binding energy in kcal/mol overall or specifically with GLU18.

| Mutation | Contribution to Binding Energy | |
|---|---|---|
| | Overall | GLU18 |
| ARG225 | -1.27 | -1.11 |
| TRP225 | -0.81 | -0.81 |
| LYS225 | -0.57 | -0.47 |
| GLN225 | -0.56 | -0.56 |
| THR225 | -0.03 | -0.03 |
| ASN225 | 0.10 | 0.10 |
| LEU225 | -0.03 | -0.03 |
| VAL225 | -0.05 | -0.05 |
| ILE225 | -0.10 | -0.10 |
| MET225 | -0.12 | -0.12 |
| CYS225 | 0.00 | 0.00 |
| SER225 | 0.00 | 0.00 |
| PRO225 | 0.00 | 0.00 |
| GLY225 | 0.00 | 0.00 |
| HIS225 | 0.00 | 0.00 |
| ALA225 | 0.00 | 0.00 |
| TYR225 | 0.00 | 0.00 |
| GLU225 | 0.07 | 0.07 |
| PHE225 | 0.00 | 0.00 |
| ASP225 | 0.13 | 0.13 |
