# Supplementary figures and images for: Ebola virus VP35 interacts non-covalently with ubiquitin chains to promote viral replication
Source: PLoS Biol. 2024 Feb 29;22(2):e3002544. doi: 10.1371/journal.pbio.3002544 (PMC10942258; doi:10.1371/journal.pbio.3002544)

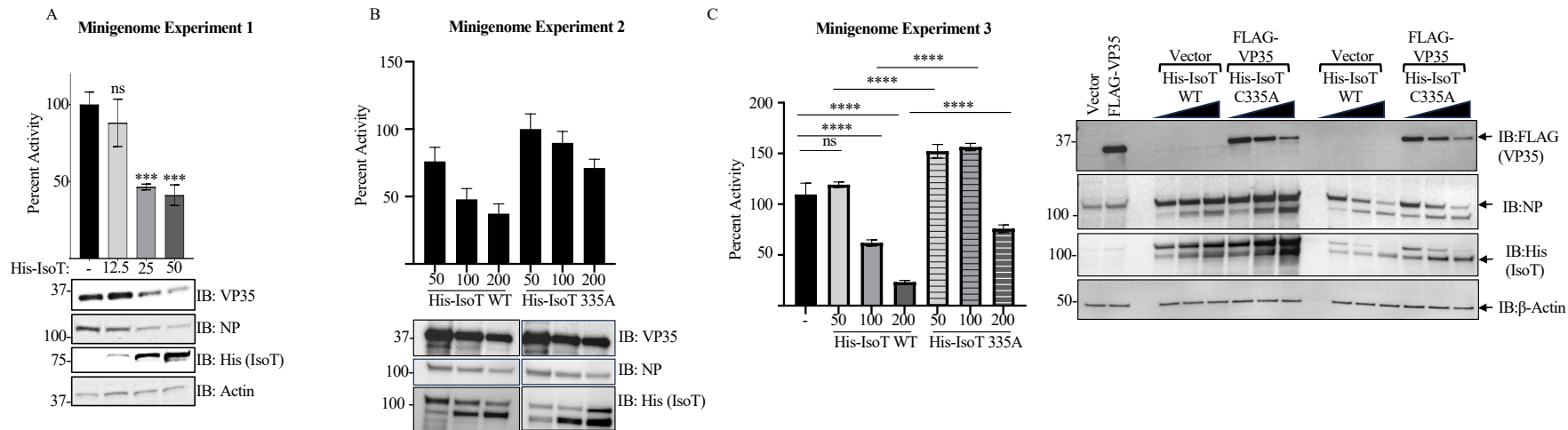

**S1 Fig.**

Supplement: S1 Fig — (A–C) HEK293T cells were transfected with minigenome components and (A) including 12.5, 25, and 50 ng of IsoT WT. (B, C) Including 50, 100, and 200 ng of IsoT WT or catalytic impaired mutant IsoT 335A, 50 h later cells were lysed for luciferase assay and western blot analysis. The data underlying the graphs shown in the figure can be found in S1 Data. (PDF) [file pbio.3002544.s005.pdf]

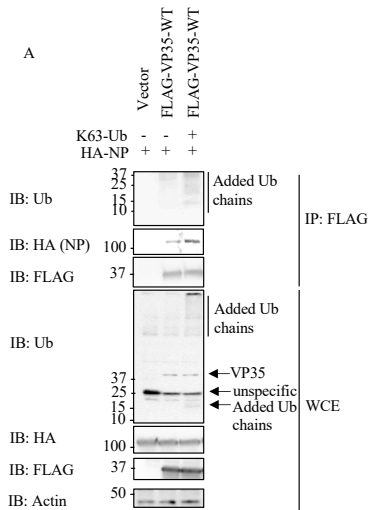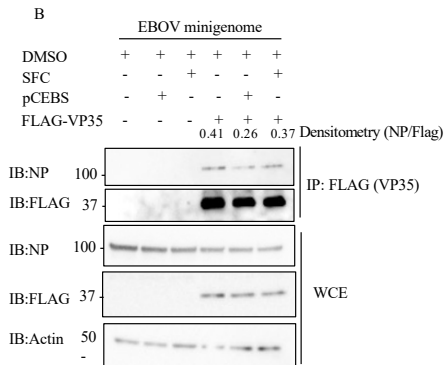

**S2 Fig**

Supplement: S2 Fig — (A) The addition of unanchored K63-linked polyUb chains enhances WT VP35 interactions with NP. Lysates from HEK293T cells expressing WT-VP35 were mixed with lysate from cells expressing NP, in the presence or absence of added purified unanchored K63-linked polyUb chains [2–12], followed by coIP with anti-Flag beads. (B) Treatment with pCEBS or SFC in a minigenome assay reduces interactions between VP35 and NP in the co-immunoprecipitation assay. HEK293T cells were transfected with minigenome components and 4 h post-transfection cells were treated with 200 μm of pCEBS and SFC; 50 h later cells were lysed, and immunoprecipitation assay was performed using FLAG beads. (PDF) [file pbio.3002544.s006.pdf]

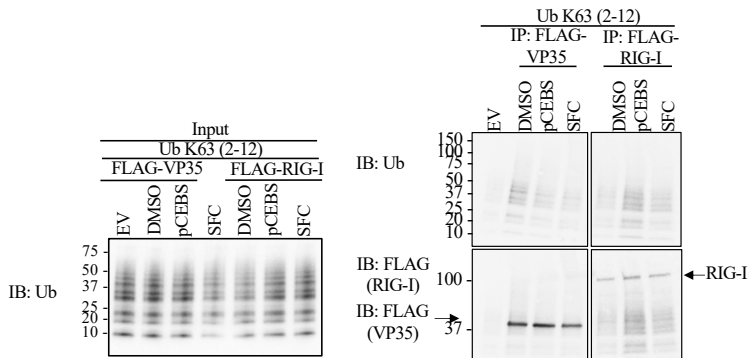

**S3 Fig**

Supplement: S3 Fig — HEK293T cells were transfected with FLAG-VP35 or FLAG-RIG-I. After lysis, FLAG immunoprecipitations were performed to isolate RIG-I and VP35, followed by incubation with purified K63-linked polyUb chains [2–12], in the presence of 200 μm or pCEBS or SFC, or DMSO as control. (PDF) [file pbio.3002544.s007.pdf]

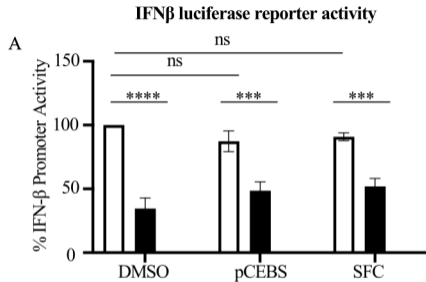

**B**

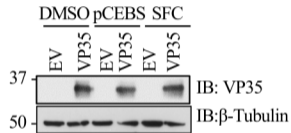

**S4 Fig**

Supplement: S4 Fig — (A) IFN-β promoter assay HEK293T cells were transfected with 100 ng of VP35 WT and treated for 24 h with 200 μm of pCEBS, SFC, or DMSO as vehicle. Then, cells were transfected with 3.125 μg/ml of HMW Poly I:C for 16 h. (B) Western blot analysis of (A). Data are depicted as mean + SEM. Two-Way ANOVA Tukey’s multiple comparisons tests. The data underlying the graphs shown in the figure can be found in S1 Data. (PDF) [file pbio.3002544.s008.pdf]

A

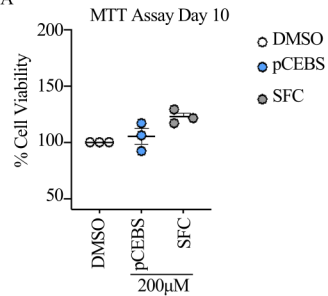

B

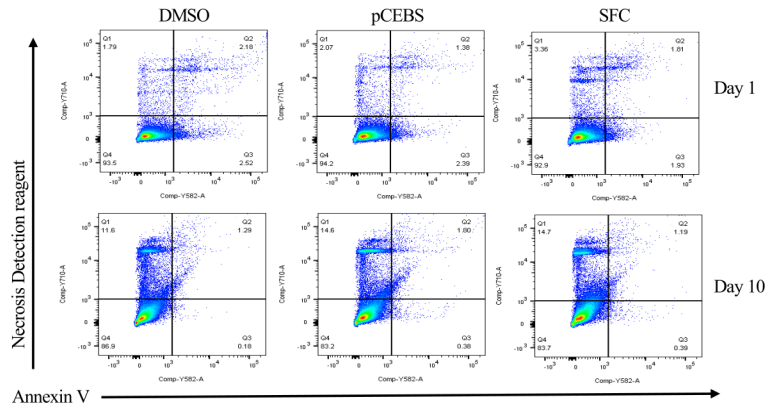

C

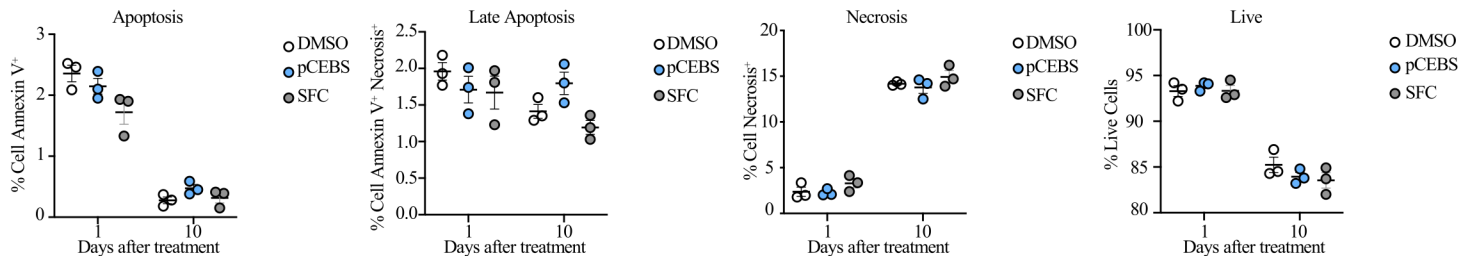

Supplement: S5 Fig — (A) Cell viability from Vero CCL81 treated with DMSO, pCEBS, or SFC 200 μm for 10 days, metabolic activity was measured using MTT assay (Thermo Fisher). (B, C) Flow cytometry analysis of Vero CCL81 treated with DMSO, pCEBS, or SFC 200 μm for 1 or 10 days, cells were then stained using ENZO GFP-Certified apoptosis/necrosis detection kit acquired in an LSR Fortessa (BD) and analyzed using flowJo. The data underlying the graphs shown in the figure can be found in S1 Data. (PDF) [file pbio.3002544.s009.pdf]

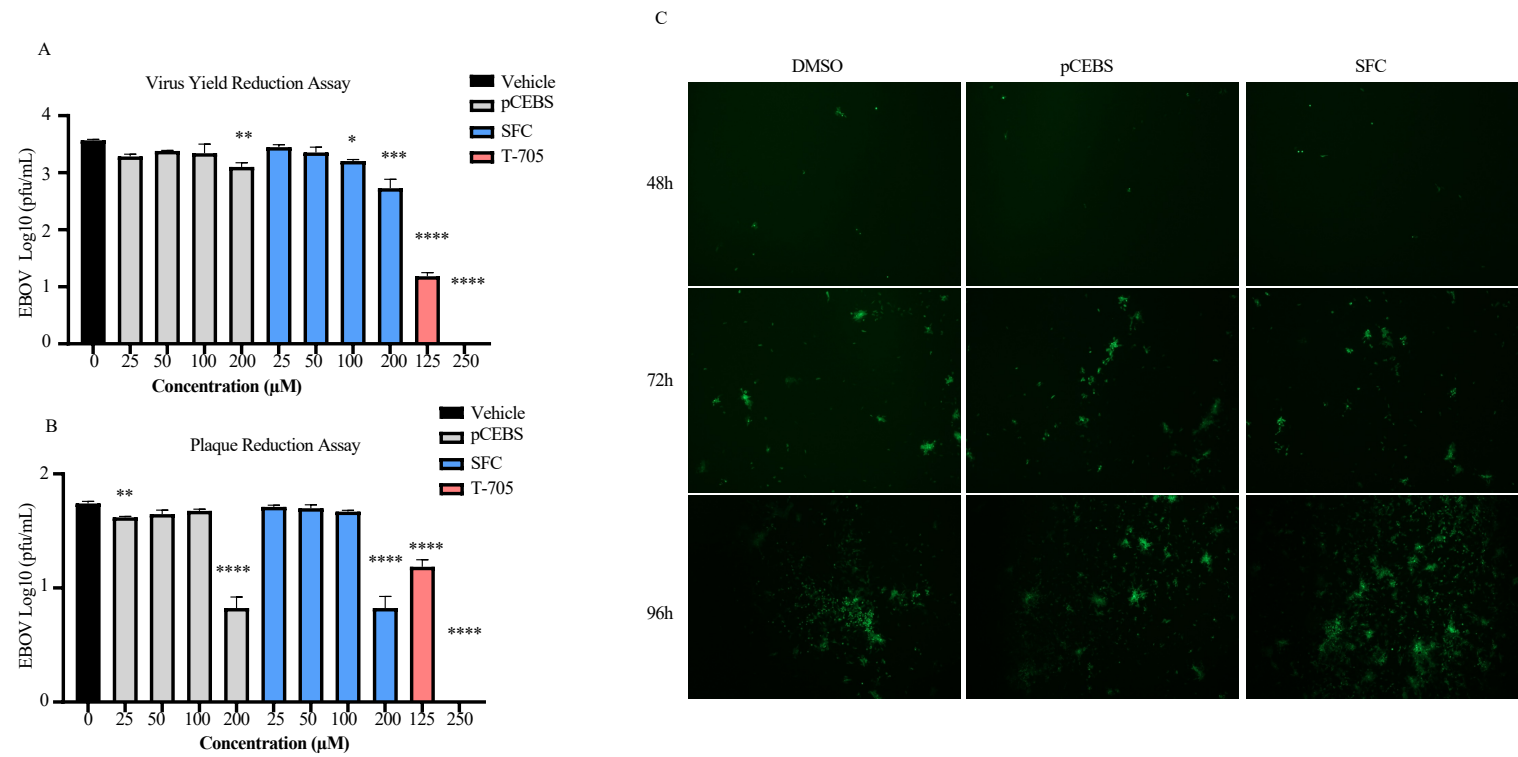

S6 Fig

Supplement: S6 Fig — (A, B) Ebola titers obtained from virus yield reduction assay expressed in Log10 by pfu/ml. (B) Ebola titers obtained from Plaque reduction assay expressed in Log10 by pfu/ml, shown in Fig 7D and 7E. Data are depicted as mean + SEM. One-Way ANOVA Tukey’s multiple comparisons test. (C) Pre-treatment with pCEBS and SFC do not affect EBOV replication. Fluorescent microscopy of Vero CCL81 cells untreated or treated with 200 μm of pCEBS or SFC for 1 h, compounds were removed, and cells were infected with EBOV-GFP MOI 0.01. The data underlying the graphs shown in the figure can be found in S1 Data. (PDF) [file pbio.3002544.s010.pdf]

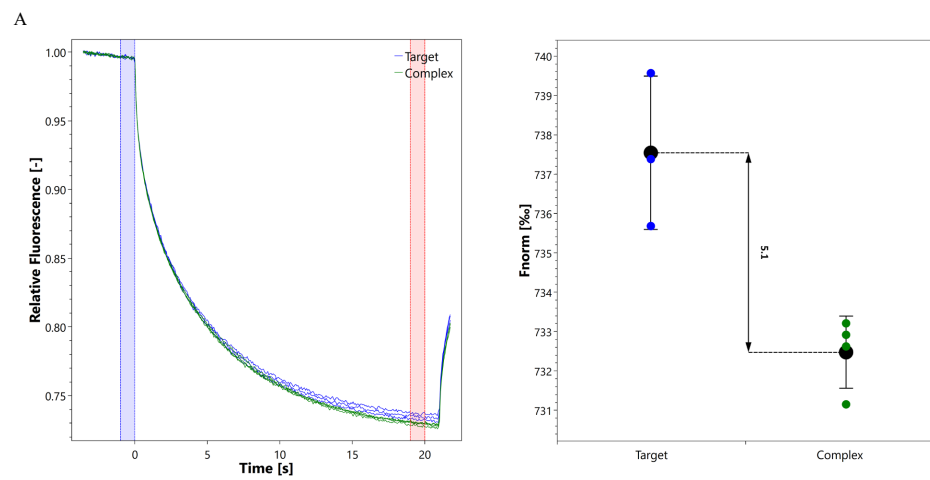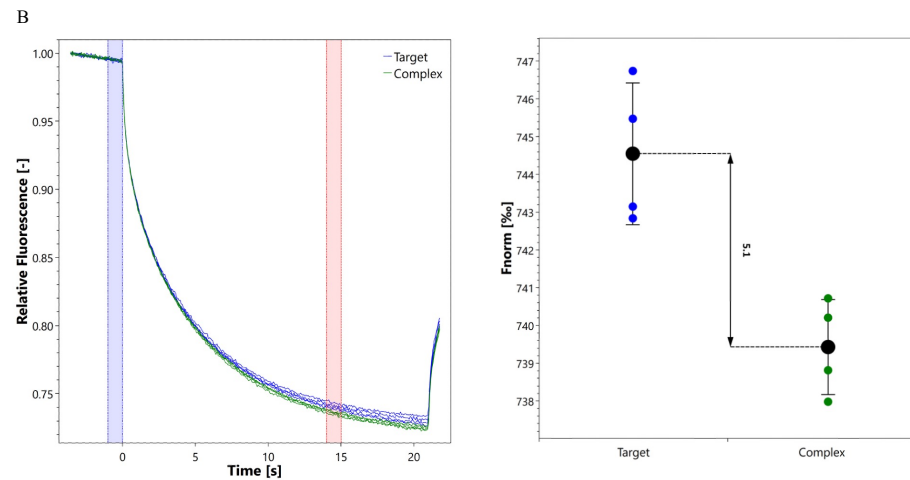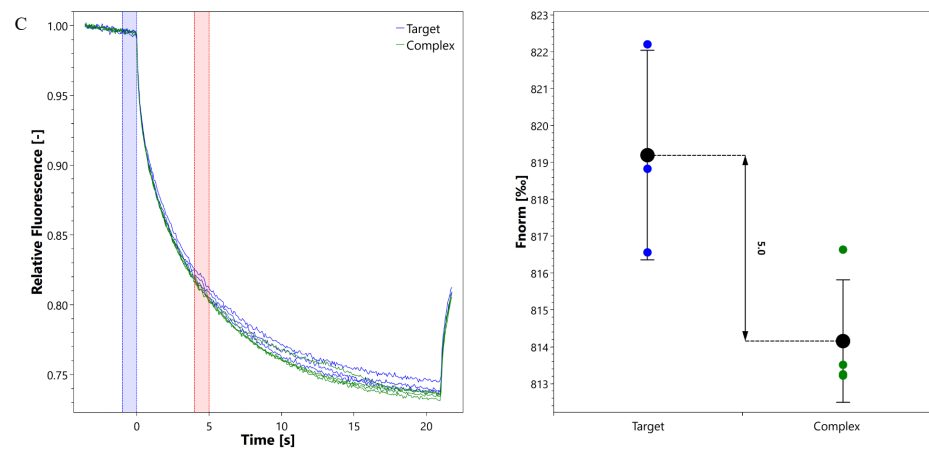

**S7 Fig**

Supplement: S7 Fig — Tagged WT VP35 in complex different interactors to determine if there are shifts normalized fluorescence (Fnorm) (A) K63-linked Ubiquitin (a mixture of n = 3–12), (B) pCEBS, and (C) SFC. The data underlying the graphs shown in the figure can be found in S1 Data. (PDF) [file pbio.3002544.s011.pdf]

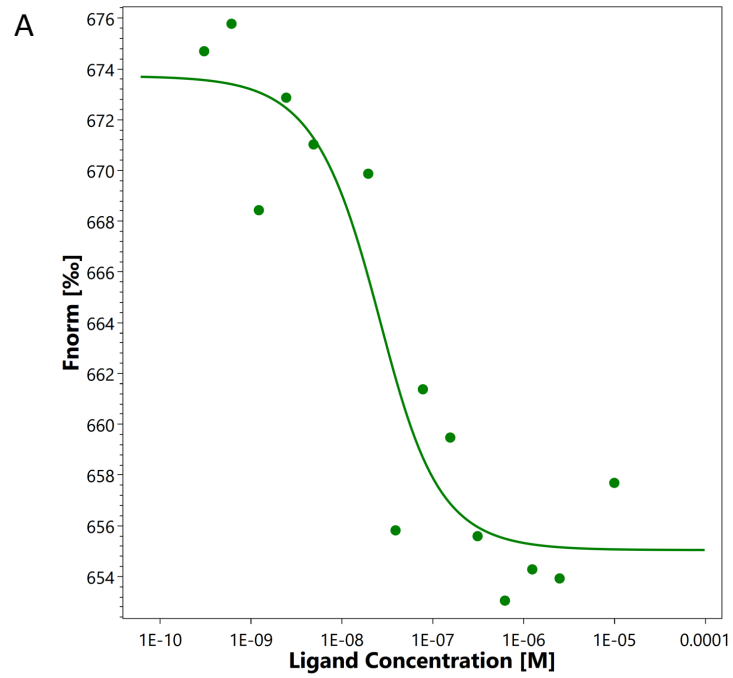

S8 Fig

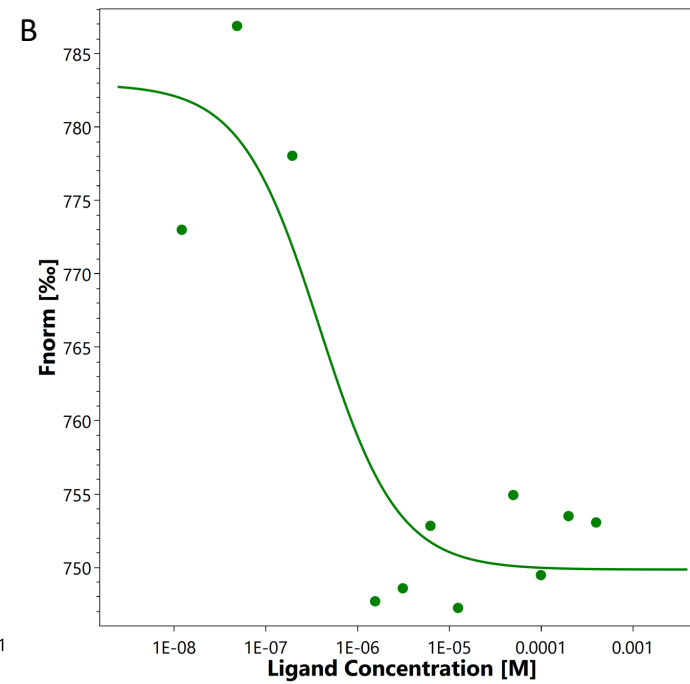

Supplement: S8 Fig — (A) Microscale thermophoresis (MST) titration curve for the VP35 Ubiquitin interaction. Normalized fluorescence (Fnorm) for Tagged WT VP35 in complex with different concentrations of Ubiquitin were measured by MST. The estimated Kd is 15 nM. (B) Microscale thermophoresis (MST) titration curve for the VP35 SFC interaction. Normalized fluorescence (Fnorm) for Tagged WT VP35 in complex with different concentrations of SFC were measured by MST. The estimated Kd is 375 nM. The data underlying the graphs shown in the figure can be found in S1 Data. (PDF) [file pbio.3002544.s012.pdf]

Ub

pCEBS

SFC

WT

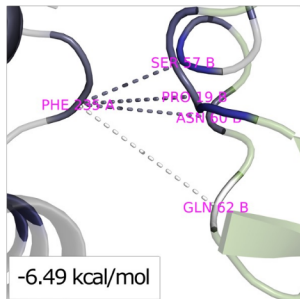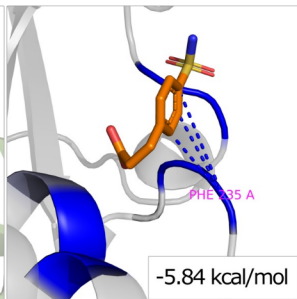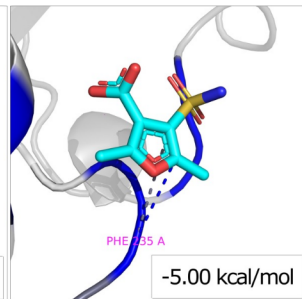

F235H

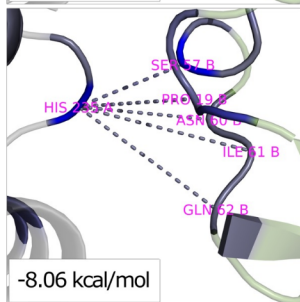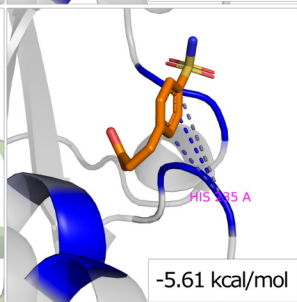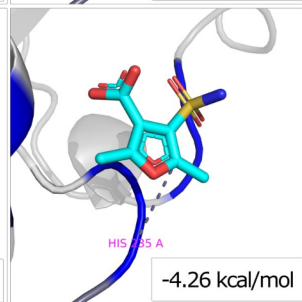

S9 Fig

Supplement: S9 Fig — Interactions between wild type (WT) VP35 protein (in gray) and the F235H mutant with ubiquitin (in green) on the left column, pCEBS (in orange, middle column), and SFC (in cyan, right column), with the dash lines representing pairwise atomic interactions that change by more than 0.1 kcal/mol between WT and F235 as predicted by Surfaces. The colors of individual dash lines, as well as the colors of entire residues, are shown in a scale of blue to white in which blue represents more favorable interactions/residues with stronger net interactions and white represents less significant interactions/residues with net interactions closer to null. Compared to WT, we see that the F235H mutation in VP35 strengthens the interaction with residues PRO19, SER57, ASN60, ILE61, and most of all with GLN62 of Ubiquitin (on the right in the 2 left-most panels). Compared to WT, the F235H mutations do not abolish interactions between this residue (H235) for either of the compounds. In both cases, however, the small molecules are predicted to have weaker interactions with residue H235 compared to F235. The overall predicted DG of binding (in kcal/mol) for each complex is displayed in each panel. The difference between the bottom and top DG values for each interaction gives a predicted DDG of −1.57 kcal/mol for the interaction with Ubiquitin, suggesting a strengthening of this interaction, whereas for pCEBS and SFC we obtain positive DDG values of 0.23 kcal/mol and 0.74 kcal/mol, respectively, suggesting that both molecules have a weaker interaction with VP35 in the F235H mutant. (PDF) [file pbio.3002544.s013.pdf]

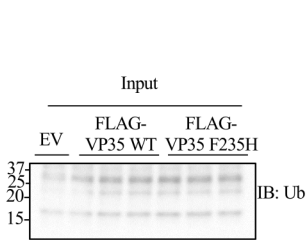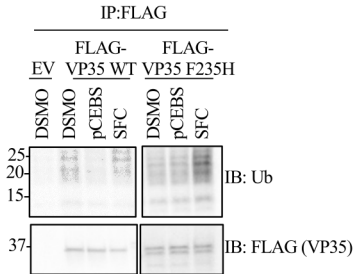

**S10 Fig**

Supplement: S10 Fig — Lysates from HEK293T cells expressing VP35 WT or VP35 F235H were incubated with anti-FLAG beads to isolate VP35. After washes, purified recombinant unanchored K63-linked polyUb chains [2–12] were incubated with the beads containing VP35 as described. After washes, Ub bound to VP35 was detected by Immunoblot (IB). (PDF) [file pbio.3002544.s014.pdf]

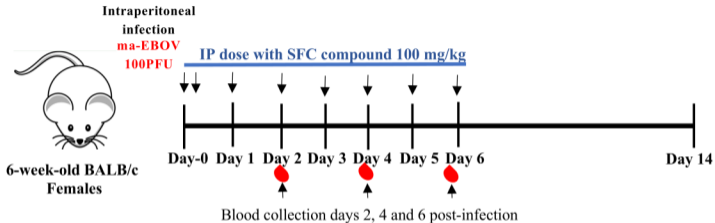

**S11 Fig**

Supplement: S11 Fig — (A) Scheme of the in vivo mouse experiment. (PDF) [file pbio.3002544.s015.pdf]
