# Supplementary material for: Ebola virus VP35 interacts non-covalently with ubiquitin chains to promote viral replication
Source: PLoS Biol. 2024 Feb 29;22(2):e3002544. doi: 10.1371/journal.pbio.3002544 (PMC10942258; doi:10.1371/journal.pbio.3002544)
Supplement: S12 Fig — Recombinant EBOV WT or VP35 K309R mutant virus was purified by sucrose gradient. Purification was performed as described in van Tol and colleagues [6]. In short, Supernatants from a T75 flasks of VeroE6 cells infected at an MOI of 0.01 PFU/cell were collected at 144 hpi for sucrose-gradient purification. The 15 ml of supernatant was first clarified to remove cellular debris before loading onto a 25% sucrose cushion. The virus was loaded onto a 20%–60% sucrose gradient and then pelleted. The pellet was resuspended in STE buffer and an aliquot was used directly for immunoblot shown in (A), as input virus. The rest of sample was used for immunoprecipitation (IP), using an anti-VP35 antibody or an IgG control, shown in (B). To obtain evidence that VP35 associates with free ubiquitin in the virion, the same amount of sample used for IP was boiled before proceeding to IP, to denature all proteins (B, right side). Ubiquitin running at high molecular weight (over 100 kDa) is dissociated from VP35, suggesting some long free ubiquitin chains are associated with VP35 non-covalently. Little difference is observed between WT and K309R viruses, suggesting that most of the ubiquitin packaged in the virion is not covalently attached on the K309 site of VP35. Aliquots of the purified virus were used for titration to demonstrate infectivity, shown in reference [6]. In panel A, based on the molecular weight of 1 unit of Ubiquitin (approximately 8.5 kDa), the purified virions contain at least free Ub and Ub chains in the form of mono-Ub, di-Ub, tri-Ub, and tetra-Ub. Longer forms of polyubiquitin chains can be detected but cannot be differentiated between free ubiquitin or covalently modified VP35, or other potential viral proteins covalently ubiquitinated. VP30, VP40, and NP are shown to demonstrate the presence of EBOV particles after the sucrose purification. (PDF) [file pbio.3002544.s016.pdf]

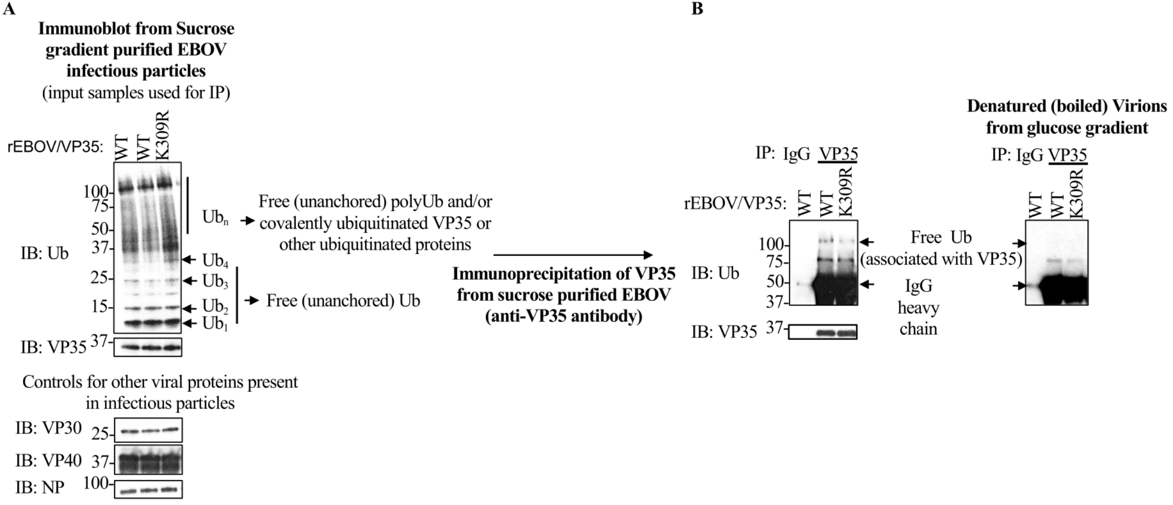
A
Immunoblot from Sucrose gradient purified EBOV infectious particles
(input samples used for IP)
rEBOV/VP35:
WT
WT
K309R
100
75
50
37
25
15
IB: Ub
Ub_n
Free (unanchored) polyUb and/or covalently ubiquitinated VP35 or other ubiquitinated proteins
Ub_4
Ub_3
Ub_2
Ub_1
Free (unanchored) Ub
IB: VP35
37
Controls for other viral proteins present in infectious particles
IB: VP30
25
IB: VP40
37
IB: NP
100
Immunoprecipitation of VP35 from sucrose purified EBOV (anti-VP35 antibody)
B
IP: IgG VP35
rEBOV/VP35:
WT
WT
K309R
IB: Ub
100
75
50
37
IB: VP35
37
Free Ub
(associated with VP35)
IgG heavy chain
Denatured (boiled) Virions from glucose gradient
IP: IgG VP35
WT
WT
K309R

S12 Fig
